# Characterization of quinoxaline derivatives for protection against iatrogenically induced hearing loss

**DOI:** 10.1172/jci.insight.141561

**Published:** 2021-03-08

**Authors:** Marisa Zallocchi, Santanu Hati, Zhenhang Xu, William Hausman, Huizhan Liu, David Z. He, Jian Zuo

**Affiliations:** Department of Biomedical Sciences, Creighton University School of Medicine, Omaha, Nebraska, USA.

**Keywords:** Neuroscience, Therapeutics, Bacterial infections, Cancer, NF-kappaB

## Abstract

Hair cell loss is the leading cause of hearing and balance disorders in humans. It can be caused by many factors, including noise, aging, and therapeutic agents. Previous studies have shown the therapeutic potential of quinoxaline against drug-induced ototoxicity. Here, we screened a library of 68 quinoxaline derivatives for protection against aminoglycoside-induced damage of hair cells from the zebrafish lateral line. We identified quinoxaline-5-carboxylic acid (Qx28) as the best quinoxaline derivative that provides robust protection against both aminoglycosides and cisplatin in zebrafish and mouse cochlear explants. FM1-43 and aminoglycoside uptake, as well as antibiotic efficacy studies, revealed that Qx28 is neither blocking the mechanotransduction channels nor interfering with aminoglycoside antibacterial activity, suggesting that it may be protecting the hair cells by directly counteracting the ototoxin’s mechanism of action. Only when animals were incubated with higher doses of Qx28 did we observe a partial blockage of the mechanotransduction channels. Finally, we assessed the regulation of the NF-κB pathway in vitro in mouse embryonic fibroblasts and in vivo in zebrafish larvae. Those studies showed that Qx28 protects hair cells by blocking NF-κB canonical pathway activation. Thus, Qx28 is a promising and versatile otoprotectant that can act across different species and toxins.

## Graphical abstract

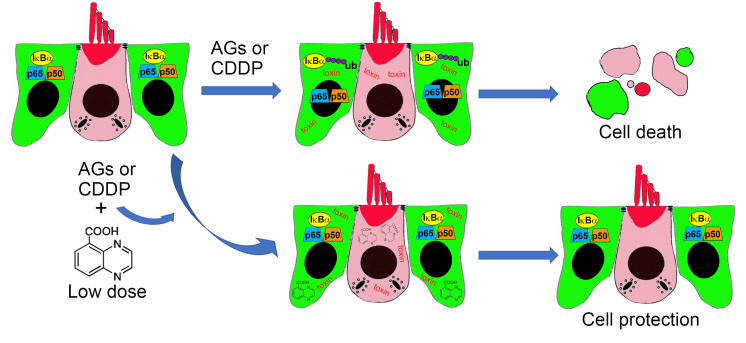

## Introduction

According to the World Health Organization (1), more than 5% of the world’s population is affected by moderate to profound hearing loss, with an estimated rise to over 900 million by 2050 if no action is taken. Moreover, the current COVID-19 outbreak may increase these estimates not only due to the possible side effects of drug treatments but also because of the viral infection (2, 3).

Hearing impairment can be caused by genetic mutations, infectious diseases, noise, aging, and exposure to ototoxic drugs (4–10). Aminoglycoside (AG) antibiotics are water-soluble molecules with potent antimicrobial properties used in the treatment of sepsis or serious opportunistic infections occurring in patients with cystic fibrosis. However, despite their clinical utility, they carry the risk of adverse side effects, including hearing loss (ototoxicity) and kidney damage (nephrotoxicity) (11–16). Nephrotoxicity occurs in approximately 60% of the patients treated with AGs. Fortunately, this damage is reversible, due to the regenerative abilities of the kidney. On the other hand, because hair cells of the inner ear do not regenerate, the damage caused by AG treatment is irreversible, resulting in hearing and balance deficits in more than 25% of the patients treated with these antibiotics (4, 17).

To date, no drugs have been approved by the US Food and Drug Administration (FDA) for protection against AG-induced hearing loss (18, 19), so there is an immediate unmet medical need for treatments. To reduce AG damage to the inner ear cochlear cells, various therapeutic strategies, including antioxidants, antiinflammatory agents, calcium channel blockers, and kinase modulators, have been used in previous studies (7, 20–25). However, clinical trials have been disappointing, possibly because the protection is not robust and disappears at higher doses of the ototoxic agent.

Recent studies have utilized large-scale drug screenings using cell lines, zebrafish lateral line hair cells, or mouse cochlear explants to identify novel otoprotective compounds (26, 27), some of which have shown better treatment efficiency than many benchmark compounds (e.g., N-acetyl cysteine, d-methionine, and dexamethasone) (8, 22, 28, 29). However, additional drugs and mechanisms of action remain to be further explored so that individual or combinatory treatment can be readily developed for complete protection against AG-induced hearing loss.

Our previous work supports the chemoprotective and therapeutic potential of quinoxaline, a nonsteroidal antiinflammatory compound, to prevent and treat AG-induced hearing loss (27). Many features of quinoxaline make it ideal as a scaffold molecule to be used in drug discovery studies: quinoxaline has an excellent malleable structure optimal for medicinal chemistry, many quinoxaline derivatives can cross the blood labyrinth barrier (BLB), and they are water-soluble and stable at physiological pH (27, 30, 31). More importantly, quinoxaline derivatives have been approved by the FDA as chemical compounds for use in the pharmaceutical and food industries (32), which would expedite their developmental phase for implementation in the auditory field as repurposed compounds (33). Based on this, and since quinoxaline only partially protected zebrafish hair cells from gentamicin and neomycin ototoxicity (27), we asked whether chemical modifications of the quinoxaline core would improve its protective effect against AG-induced hair cell death. For this purpose, we synthesized a library of quinoxaline derivatives and screened them in a zebrafish line that expresses a hair cell membrane-bound green fluorescence protein (27, 34). The screening results identified quinoxaline-5-carboxylic acid (Qx28) as one of the top-hit candidates exhibiting excellent protection (approximately 50% more than quinoxaline) against acute and long-term AG-induced hair cell loss. Moreover, Qx28 protects against AG-induced hair cell loss in mouse cochlear explants. Studies in mouse embryonic fibroblasts (MEFs), zebrafish morphants, and the reporter zebrafish line *NFKB:EGFP* (35) suggest that the NF-κB canonical pathway is one of the main signaling cascades targeted by Qx28 in response to ototoxic insult.

Taken together, the present study provides a proof of concept for our screening platform strategy aimed at the identification of new and repurposed drugs for protection against different types of hearing loss.

## Results

### Screening of a quinoxaline derivative library for protection against AG-induced ototoxicity.

There are many aspects of zebrafish that make it a useful model for the screening of small molecules and natural compounds to identify regulators of hair cell survival, regeneration, and toxicity. They are highly fertile, optically transparent, and small, which facilitates high-throughput drug screenings by the addition of the compounds of interest into the media (36–38). Furthermore, since zebrafish hair cells are morphologically and functionally similar to their mammalian counterparts and respond similarly to ototoxic insults, any potential compound exerting a beneficial effect in fish is likely to have a similar effect in mammals (36–40). We previously found that while quinoxaline treatment fully protected neuromast hair cells from cisplatin (CDDP) ototoxicity, it performed poorly against neomycin and the long-term deleterious effect of gentamicin (27). Given this, we chemically modified quinoxaline’s core to identify derivatives that will better protect against aminoglycoside ototoxicity.

Since quinoxaline is a privileged scaffold molecule (41), we explored its chemical space and tested the effect of various substitutions at different positions (Supplemental Table 1; supplemental material available online with this article; https://doi.org/10.1172/jci.insight.141561DS1). Because of its symmetrical structure, we incorporated a single methyl group at positions 5 and 6 or multiple methyl groups at positions 2, 3, and 6 of quinoxaline’s benzene ring (i.e., Qx42, Qx68, and Qx3). By introducing these methyl groups, we were expecting to increase the lipophilicity of quinoxaline and to drastically change its bioavailability. We also assessed the functional consequences of including additional groups at position 2 of the quinoxaline ring. Halogens, methyl-ester, carboxamide, hydroxy, and carboxylic acid were incorporated to test the steric and electronic effects on the quinoxaline scaffold. While esters can change the solubility properties of a compound due to their electrophilicity, halogens can act as Lewis bases or acids. Moreover, the presence of halogen groups in drug discovery is very common (42); approximately 40% of the clinical trial and commercially available drugs are halogenated (43). To increase water solubility, we inserted a carboxylic acid group at different positions (i.e., Qx28 and Qx66). Since acidic and basic functional groups are capable of ionization, they can negatively or positively charge the molecule, increasing its potency. Changes in the position of the carboxamide, hydroxy, amino, and carboxylic acid groups can also change hydrogen bonding and water solubility. Additionally, we explored substitutions at 5 and 6 positions to enrich our library with functionalities such as chloromethyl, amino, nitro, and ethyl ester. When considering the nitro group, it is one of the most versatile functional groups that possesses a strong electron attracting capability. Nitro groups can create localized electron-deficient sites within molecules, modifying the way compounds interact with their biological targets. The substitutions of the quinoxaline core with a thiol or a methoxy group were designed to increase the donation of electrons to the aromatic ring and, thus, the electron availability of quinoxaline’s nitrogen atom. We also used bioisosteric replacement of the hydrogen atom present in the methyl and methoxy groups to test whether we could modify quinoxaline activity without any increase in toxicity (i.e., Qx8, Qx49, and Qx60). To assess the effect of di-substitutions on the quinoxaline ring, we explored simultaneous substitutions at positions 2 and 3 with methoxy, amino, and chloro as the functional groups (i.e., Qx21, Qx23, and Qx50). Finally, to test whether different functionalities can increase the protection ability of quinoxaline, we incorporated tri- and tetra-substitutions at multiple positions of the core structure to broaden the scope of our library (i.e., Qx48 and Qx56).

**Figure 1:**
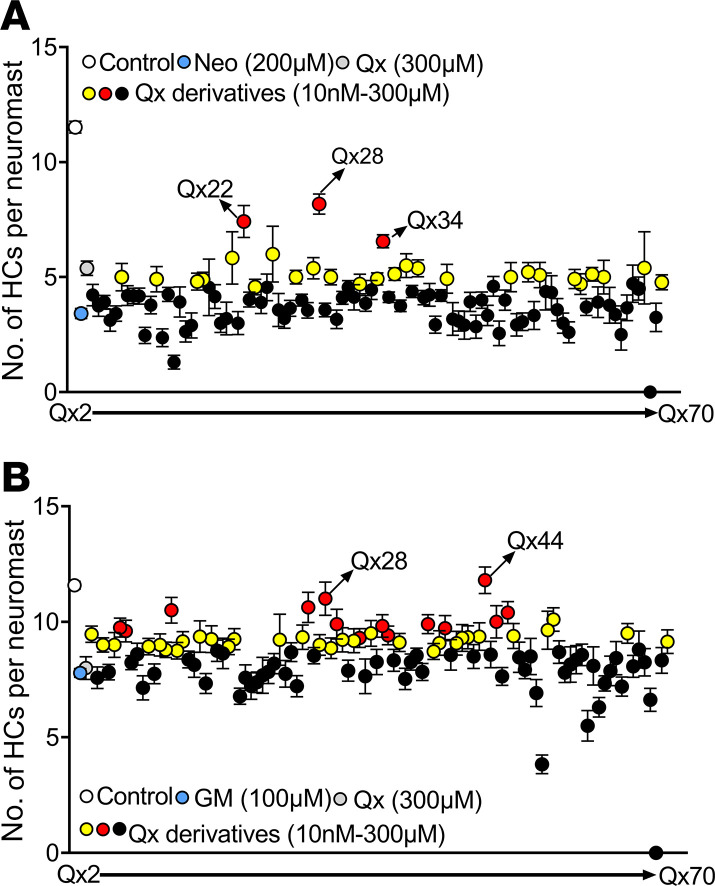
Screening of quinoxaline derivatives. Five dpf *Tg(brn3c:GFP)* zebrafish were preincubated with quinoxaline (Qx, 300 μM) or its derivatives (Qx2–Qx70, 10 nM–300 μM) for 1 hour, followed by coincubation with neomycin (Neo) 200 μM for 30 minutes (**A**) or gentamicin (GM) 100 μM long-term effect (**B**). Hair cells were quantified employing a Zeiss AxioSkop 2 fluorescence microscope with a 40× oil objective. White dot, vehicle; blue dot, ototoxin alone; gray dot, quinoxaline 300 μM. Black dots, quinoxaline derivatives that did not show any significant differences compared with ototoxin alone. Yellow dots, quinoxaline derivatives that performed significantly better than ototoxin alone but not significantly different from quinoxaline treatment. Red dots, quinoxaline derivatives that performed significantly better than quinoxaline treatment. Results were expressed as mean ± SEM. Statistical analysis: 1-way ANOVA with correction for Dunnett’s multiple comparisons test. Significance was set at *P* < 0.05 versus ototoxin or quinoxaline. Six fish were used per treatment, and 3 neuromasts were inspected per fish (*n* = 18).

By doing the chemical modifications described above, we generated a library of 68 quinoxaline derivatives that were tested against AG-induced ototoxicity at the initial dose that was used in our previous quinoxaline studies (27). Five days postfertilization (dpf) *Tg(brn3c:GFP)* larvae (27, 34) were incubated with the ototoxin alone or in the presence of one of the derivatives and the number of hair cells per neuromast counted and compared with control and ototoxin alone (Figure 1). Those compounds showing similar or better levels of protection than quinoxaline (27) were, then, tested at lower doses until there were no significant differences between the derivative treatment and neomycin or gentamicin alone (Figure 1 and Supplemental Table 1).

Out of that diverse library, only 3 derivatives, Qx22, quinoxaline-5-carboxylic acid (Qx28), and Qx34, performed better than quinoxaline during neomycin treatment (Figure 1A). While several derivatives performed better than quinoxaline during long-term gentamicin ototoxicity, Qx28 and Qx44 showed the best results (Figure 1B). Surprisingly, the compounds with methyl or halo substitution did not improve quinoxaline’s activity.

Because treatment with quinoxaline-5-carboxylic acid (Qx28) resulted in protection against both types of AG ototoxicity, we focused our subsequent studies on this particular quinoxaline derivative.

### Qx28 protects against AG- and CDDP-induced hair cell death with excellent efficacy and potency.

**Figure 2:**
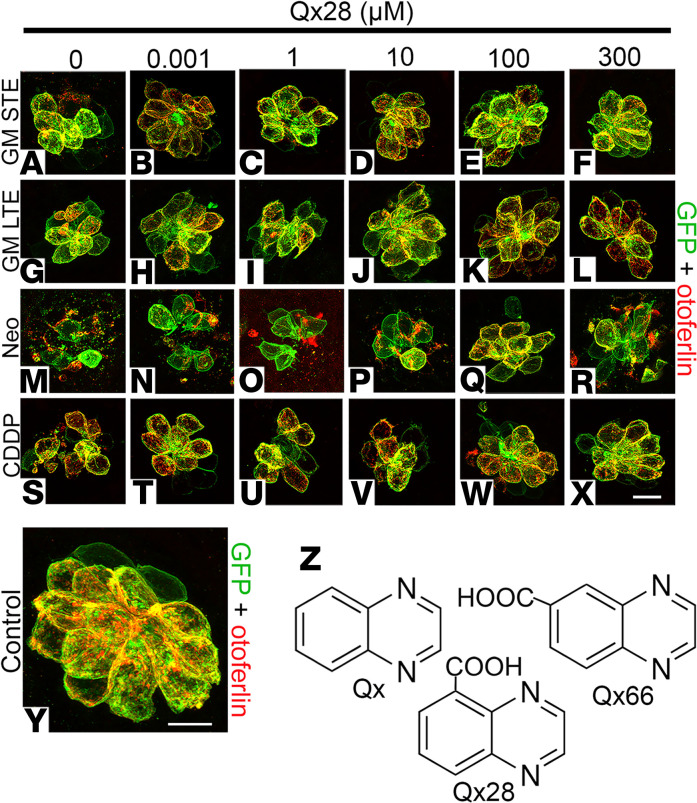
Qx28 treatment in neuromast hair cells. Representative images of 5 dpf *Tg(brn3c:GFP)* zebrafish pretreated with Qx28 (1 nM–300 μM) followed by coincubation with the different ototoxins. (**A**–**F**) Short-term effect (STE) of gentamicin 100 μM for 1 hour followed by 1-hour recovery in E3 water. (**G**–**L**) Long-term effect (LTE) of gentamicin 100 μM for 1 hour followed by 5-hour recovery in E3 water. (**M**–**R**) Neomycin 200 μM for 30 minutes followed by 1-hour recovery in E3 water. (**S**–**X**) CDDP 400 μM for 6 hours followed by 1-hour recovery. (**Y**) Vehicle control neuromast. GFP shown in green, otoferlin shown in red. Neuromasts were imaged employing a Zeiss LSM 800 confocal microscope with a 63× oil objective and Airyscan function. Scale bars: 7 μm (**A**–**X**), 5 μm (**Y**). (**Z**) Chemical structure of Qx, Qx28, and Qx66. For sample numbers see legend of Figure 3.

**Figure 3:**
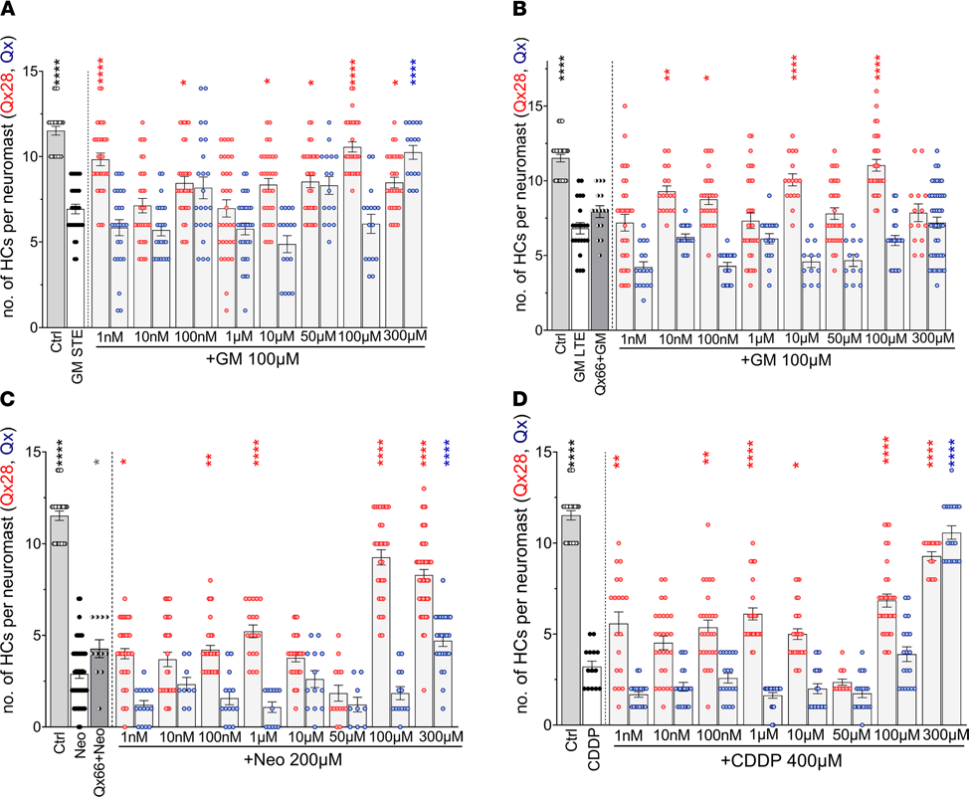
Qx28 protects from aminoglycoside- and CDDP-induced hair cell death. Quantification of the number of hair cells per neuromasts after the different ototoxin treatments in the presence or absence of quinoxaline (Qx, blue dots) or Qx28 (red dots). (**A**) Short-term gentamicin exposure (100 μM). (**B**) Long-term gentamicin exposure (100 μM). (**C**) Neomycin exposure (200 μM). (**D**) CDDP exposure (400 μM). Qx66 50 μM was included to show that although it has a similar structure as Qx28, it does not protect. Results are expressed as mean ± SEM. Statistical analysis: 1-way ANOVA with correction for Dunnett’s multiple comparisons test. *P < 0.05, **P < 0.01, ****P < 0.0001 versus ototoxin alone (blue asterisks, quinoxaline treatment; red asterisks, Qx28 treatment). Neuromasts inspected: vehicle = 20; GM-STE: GM alone = 29; GM+Qx28 = 29 (1 nM, 300 μM), 31 (10 nM–100 μM); GM+Qx = 25 (1 nM–100 nM), 30 (1 μM), 16 (10 μM–300 μM). GM-LTE: GM alone = 22; GM + Qx66 = 15; GM+Qx28 = 30 (1 nM, 1 μM, 50 μM, 100 μM), 17 (10 nM, 10 μM), 25 (100 nM), 14 (300 μM); GM+Qx = 17 (1 nM–1 μM, 100 μM), 12 (10 μM, 50 μM), 42 (300 μM). Neo: Neo alone = 44; Neo+Qx66 = 11; Neo+Qx28 = 28 (1 nM, 100 μM), 22 (10 nM, 100 nM, 10 μM), 18 (1 μM), 13 (50 μM), 34 (300 μM); Neo+Qx = 4 (1 nM, 100 nM, 10 μM, 100 μM), 9 (10 nM, 50 μM), 12 (1 μM), 24 (300 μM). CDDP: CDDP alone = 14; CDDP + Q28 = 19 (1 nM, 1 μM), 27 (10 nM, 100 nM), 23 (10 μM), 10 (50 μM), 32 (100 μM), 14 (300 μM); CDDP+Qx = 19 (1 nM–100 nM, 50 μM–300 μM), 13 (1 μM), 15 (10 μM).

CDDP is a platinum-based drug widely used as a chemotherapeutic agent to treat various types of cancers (44). Similar to AG treatment, 2 of its major side effects are nephrotoxicity and sensorineural hearing loss (4, 45), with around 50% of the CDDP-treated patients experiencing significant and irreversible hearing impairment (44–47). Based on this information we assessed whether Qx28 can protect not only against AGs but also against CDDP in dose responses. For this purpose, 5 dpf zebrafish were incubated with gentamicin, neomycin, or CDDP in the presence or absence of different concentrations of Qx28 (Figure 2 and Figure 3). For comparison purposes, experiments with the original quinoxaline molecule were run in parallel with Qx28 (Figure 3). When animals were treated with gentamicin to assess its STE (Figure 2, A–F, and Figure 3A), Qx28 showed significant protection at most of the concentrations tested. Moreover, Qx28 performed better than quinoxaline at all the concentrations except for the highest one (300 μM), showing significant protection at 1 nM, 100 nM, and 10 μM to 300 μM. Qx28’s efficacy and potency were higher than quinoxaline (Figure 3A). We were able to achieve full protection against gentamicin’s STE when zebrafish were treated with 1 nM of Qx28; this is approximately 100,000 times less than quinoxaline’s concentration. When analyzing otoprotection against gentamicin’s LTE, quinoxaline treatment (300 μM) failed to protect hair cells from the lateral line (Figure 3B). Conversely, Qx28 treatment at concentrations ranging from 10 nM to 100 μM resulted in significant protection against gentamicin’s LTE (Figure 2, G–L, and Figure 3B). The exceptions were Qx28 1 μM and 50 μM, for which, although there was a trend, the results were not significant compared with gentamicin alone. The combination of gentamicin and 300 μM of Qx28 was toxic to the fish. Animals cotreated with neomycin and Qx28 showed a reduction in hair cell loss (Figure 2, M–R, and Figure 3C). At lower concentrations, the protective effect was modest but significant and similar to quinoxaline at higher concentrations. When zebrafish were incubated with Qx28 at concentrations of 100 μM or higher, approximately 80% of the hair cells were still present in the inspected neuromasts, demonstrating that Qx28 is a better otoprotectant than quinoxaline.

We previously characterized quinoxaline as an excellent compound for protection against CDDP-induced hair cell loss (27). When comparing both quinoxaline and Qx28, although Qx28’s efficacy against CDDP-induced hair cell loss was lower than quinoxaline (approximately 100% recovery versus approximately 73% recovery at 300 μM), its potency was much higher. Qx28 was protective at doses that were 100,000 times lower than quinoxaline (Figure 2, S–X, and Figure 3D).

In conclusion, when comparing Qx28 to quinoxaline, we can say that it is more efficacious against AG ototoxicity and more potent against both AG- and CDDP-induced hair cell damage. These properties are given by the presence of a carboxylic acid in position 5 of quinoxaline’s chemical core (Figure 2Z). The position of the carboxylic acid is also important since Qx66, which has the same functional group but in position 6, showed marginal or no protection when tested at its optimal dose of 50 μM (Figure 3, B and C). Thus, a single substitution of a carboxylic acid group at positions 2 (Qx12), 5 (Qx28), or 6 (Qx66) of the quinoxaline core (Figure 1 and Figure 2) showed a wide difference in activity, with the substitution at position 5 improving quinoxaline’s efficacy and potency.

**Figure 4:**
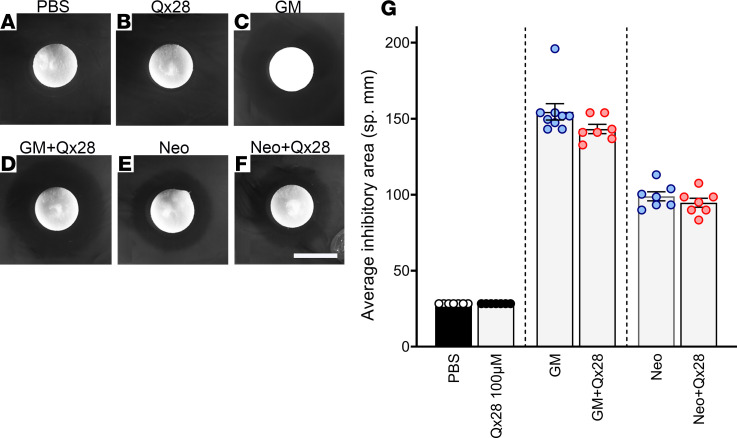
Antibiotic efficacy in the presence of Qx28. (**A**–**F**) There were no significant differences in the bacterial growth–inhibitory areas between aminoglycoside alone (**C** and **E**) (10 μg/mL) or Qx28 at the highest concentration (100 μM) (**D** and **F**). Filters soaked in PBS (**A**) or Qx28 alone (**B**) did not show any inhibitory properties. Quantification of the inhibitory area (**G**) is expressed as mean ± SEM (*n* = 9 for GM, *n* = 7 for the additional conditions). Two-tailed Student’s *t* test analysis between aminoglycoside alone or in combination with Qx28. Scale bar: 6 mm.

Because this otoprotective effect can be just the result of an interaction between Qx28 and the AGs, we decided to test whether Qx28 interferes with gentamicin and neomycin antibiotic activity. For this purpose, we performed a disc diffusion test (27) in which WT bacteria (*E. coli* strain ATCC25922) were exposed to a minimum inhibitory concentration of each antibiotic (10 μg/mL) alone or in the presence of a high dose of Qx28 (100 μM), and the inhibitory area was calculated after overnight incubation (Figure 4). Results from these experiments confirmed that Qx28 did not affect the ability of AGs to inhibit bacteria growth (Figure 4G). There were no significant differences between gentamicin and neomycin alone (Figure 4, C and E) or with Qx28 (Figure 4, D and F), suggesting Qx28 is directly protecting the hair cells from AG toxicity and not through antibiotic inactivation.

### Analysis of AG’s uptake in the presence of Qx28.

**Figure 5:**
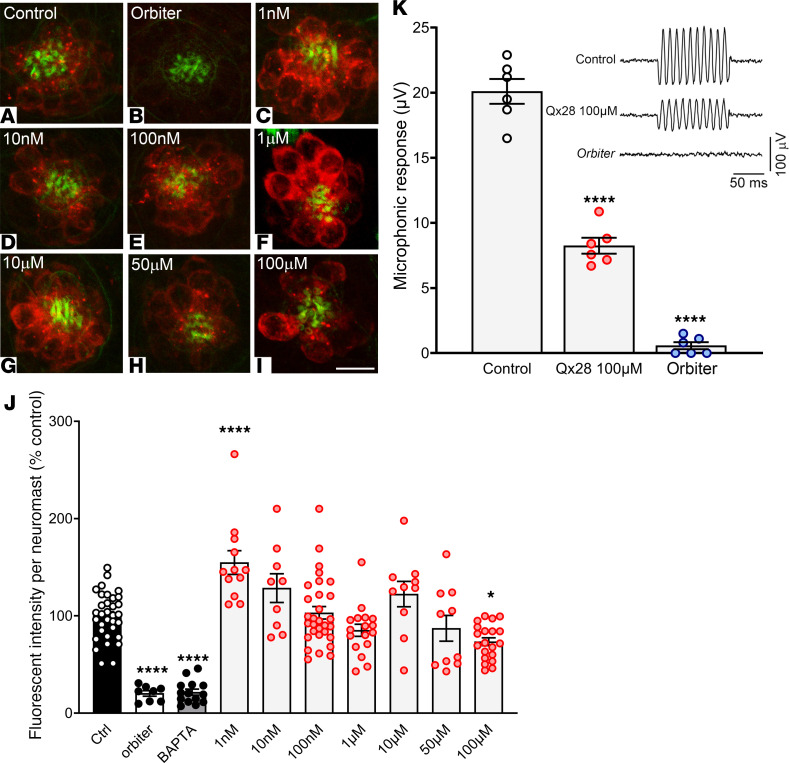
Hair cell activity in the presence of Qx28. (**A**–**I**) Five dpf WT larvae were preincubated in E3 water without (**A** and **B**) or with different Qx28 concentrations (**C**–**I**) for 1 hour and then cotreated with FM1-43 3 μM for 20 seconds. (**J**) The fluorescence incorporated per neuromast (red) was measured using ImageJ (NIH) and expressed as a percentage from control. The *orbiter* line (**B**) (52, 53) and fish preincubated with BAPTA were used as negative controls. Phalloidin counterstaining is shown in green. Results are expressed as mean ± SEM. Statistical analysis: 1-way ANOVA with correction for Dunnett’s multiple comparisons test. *P < 0.05, ****P < 0.0001 versus control. Number of neuromasts quantified: control = 33, *orbiter* = 8, BAPTA = 14, Qx28 1 nM = 12, Qx28 10 nM = 9, Qx28 100 nM = 30, Qx28 1 μM = 17, Qx28 10 μM and 50 μM = 10, Qx28 100 μM = 20. Scale bar: 7 μm. (**K**) Means ± SEM of the magnitude of microphonic responses from 6 dpf zebrafish in the presence of vehicle or Qx28 100 μM. Statistical analysis: Student’s 2-tailed *t* test. ****P < 0.0001 versus control. Measurements were obtained from 1 neuromast per fish from a total of 6 fish per condition. Top right: Microphonic potential traces of 6 dpf zebrafish in the presence of vehicle or Qx28 100 μM. The *orbiter* line was used as a negative control.

Previously published work suggests that AGs’ entry into the hair cells is mechanotransduction (MET) channel dependent and requires endocytic activity (38, 40, 48–51). Based on this assumption, any compound that, for example, blocks the MET channels will also block the entrance of these ototoxins and therefore protect against their deleterious effect. To test whether Qx28 confers protection by MET channel blockage, we performed FM1-43 uptake experiments in which fish were exposed to the dye for a very short time (27, 49, 50). Zebrafish larvae were preincubated with different concentrations of Qx28 for 1 hour and then coincubated with 3 μM of FM1-43 for 20 seconds (Figure 5). These experiments showed a rapid entry of the dye into the hair cells even in the presence of higher Qx28 concentrations (Figure 5, A–I). Quantification of the fluorescence intensity incorporated per neuromast did not show any significant decrease at low and intermediate Qx28 concentrations (Figure 5J). One exception was the dose of 100 μM, for which Qx28 partially reduced dye uptake (Figure 5, I–J). Moreover, at some concentrations, Qx28 increased dye uptake, suggesting a facilitation mechanism. We included 2 negative controls for FM1-43 uptake: (a) a genetic model (*orbiter^th263b^*) with splayed hair cell bundles due to mutations in Protocadherin-15a (*Pcdh15a*) (Figure 5, B and J, refs. 52, 53) and (b) a pharmacological model in which the tip links were disrupted by preincubating the animals with 10 mM of 1,2-bis(o-amino phenoxy)ethane-N,N,N′,N′-tetraacetic acid) (BAPTA) (Figure 5J, ref. 53). As expected, both the *Pcdh15a* mutants and the BAPTA-treated animals showed low levels of FM1-43 incorporation because of reduction of MET channel open probability (51, 53).

To confirm the partial MET channel blockage at the highest Qx28 dose, we performed microphonic potentials in 6 dpf zebrafish exposed to E3 media alone or in the presence of 100 μM of Qx28 (Figure 5K). Results from these experiments showed a decrease in the magnitude of the microphonic responses when Qx28 was present at higher concentrations. In contrast, the *orbiter* zebrafish line that lacks functional hair cells did not show microphonic responses. These results, with the FM1-43 uptake experiments, suggest that the main mechanism for Qx28 otoprotection is not through MET inhibition since Qx28 was protective at concentrations that allow dye incorporation.

**Figure 6:**
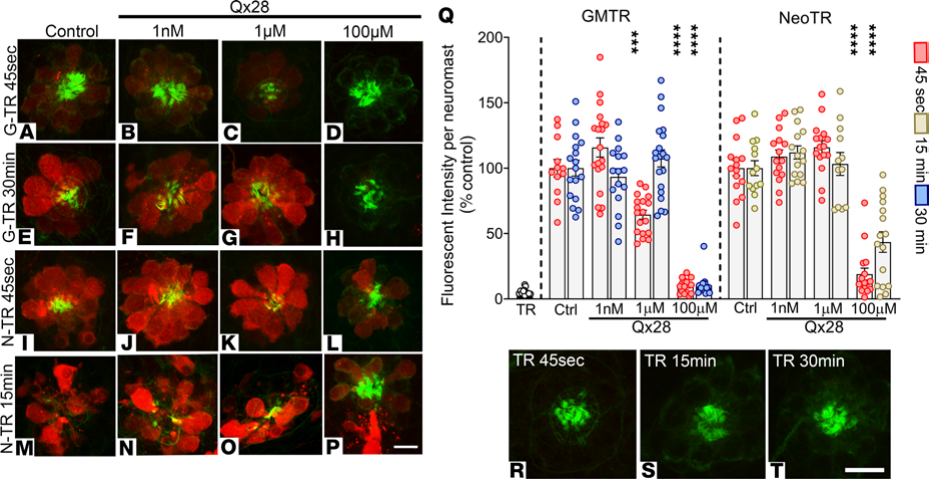
AG uptake in the presence of Qx28. Five dpf zebrafish were incubated with Texas Red–conjugated AG (AGTR) in the absence or presence of Qx28 (1 nM–100 μM). AGTR incorporation was followed over time. (**A**–**H**) Fish incubated for 45 seconds (**A**–**D**) or 30 minutes (**E**–**H**) with 50 μM of Texas Red–conjugated gentamicin (GMTR) in E3 media alone (**A** and **E**) or with Qx28 1 nM (**B** and **F**), 1 μM (**C** and **G**), or 100 μM (**D** and **H**). (**I**–**P**) Fish incubated for 45 seconds (**I**–**L**) or 15 minutes (**M**–**P**) with 100 μM of Texas Red conjugated neomycin (NeoTR) in E3 media alone (**I** and **M**) or with Qx28 1 nM (**J** and **N**), 1 μM (**K** and **O**), or 100 μM (**L** and **P**). Fish were counterstained with phalloidin (green). (**Q**) The fluorescence intensity incorporated was calculated using ImageJ and expressed as a percentage from the corresponding control without Qx28. (**R**–**T**) Texas Red (TR) incubation for 45 seconds (**R**), 15 minutes (**S**), or 30 minutes (**T**). Results are expressed as mean ± SEM. Statistical analysis: 1-way ANOVA with correction for Dunnett’s multiple comparisons test. ***P < 0.01, ****P < 0.0001 versus the corresponding control. Number of neuromasts quantified per treatment: GMTR 45 seconds = 12 (alone), 18 (+Qx28 1 nM, 1 μM), 16 (+Qx28 100 μM); GMTR 30 minutes = 17 (alone), 16 (+Qx28 1 nM), 20 (+Qx28 1 μM), 13 (+Qx28 100 μM); NeoTR 45 seconds = 15; NeoTR 15 minutes = 12 (alone, +Qx28 1 μM), 15 (+Qx28 1 nM), 16 (+Qx28 100 μM). Scale bars: 8 μm (**A**–**P**), 9 μm (**R**–**T**).

Finally, to assess the entry of the AGs into the hair cells in the presence of different Qx28 concentrations, we used Texas Red–conjugated AGs (AGTRs) and followed them over time. The use of fluorescent AGs as proxies to study AGs’ behavior is a well-established approach that allows the visualization of their subcellular distribution and accumulation (27, 51). We conjugated gentamicin and neomycin to Texas Red (GMTR and NeoTR, respectively) and exposed 5 dpf fish to a low dose of these AGTRs for a short period to reduce hair cell death due to AG ototoxicity (Figure 6). As expected from the FM1-43 uptake results, incubation with Qx28 100 μM resulted in a significant decrease in the AGTR uptake (Figure 6, D, H, L, P, and Q). At this Qx28 concentration, the AG uptake was minimal and very similar to Texas Red alone (Figure 6, R–T). Conversely, when fish were incubated with 1 μM or 1 nM of Qx28 (Figure 6, B, C, F, G, J, K, N, O, and Q), 2 otoprotective doses, the AGTR was incorporated into the hair cells, demonstrating that Qx28’s protective effect is not the result of inhibition in AG’s uptake but rather an interference with their intrinsic deleterious mechanism of action.

Taken together, these results suggest that Qx28 protects hair cells intracellularly, more likely by interfering with the ototoxin’s activity, and that MET channel activity is probably not playing a major role at low and intermediate Qx28 doses.

### Qx28 protects against gentamicin- and CDDP-induced hair cell loss in cochlear explants.

**Figure 7:**
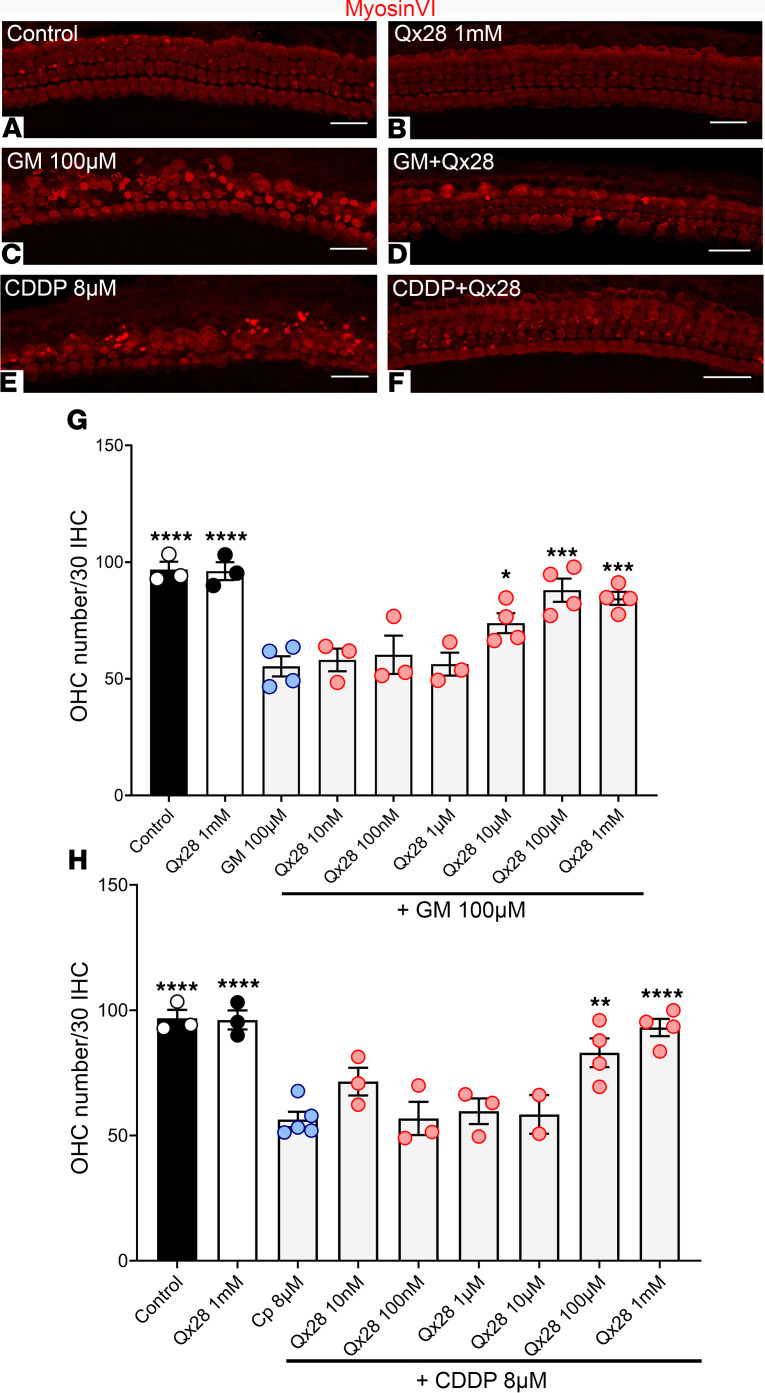
Qx28 protects mammalian hair cells from gentamicin- and CDDP-induced ototoxicity. (**A**–**H**) P3 cochlear mouse explants were incubated for 48 hours at 37°C with media (**A**), Qx28 1mM (**B**), and the ototoxins alone (**C** and **E**) or in combination with Qx28 (10 nM–1 mM) (**D** and **F**–**H**). Tissue was immunostained for myosin VI (red). Two hundred micrometer regions from the middle turn were photographed, and the number of intact outer hair cells (OHCs) in each 30 inner hair cells was counted (**G** and **H**). Results are expressed as mean ± SEM. Statistical analysis: 1-way ANOVA with correction for Dunnett’s multiple comparisons test. *P < 0.05, **P < 0.01, ***P < 0.001, and ****P < 0.0001 versus the corresponding ototoxin. Number of independent replicates: *n* = 4 (GM 100 μM, GM+Qx28 10 μM–1 mM, CDDP 8 μM, CDDP+Qx28 100 μM, 1 mM), *n* = 3 (additional treatments). Scale bar: 20 μm.

We next sought to establish whether Qx28 can protect mammalian hair cells from ototoxic insult. For this purpose, we employed neonatal mouse cochlear explants as our experimental platform to test Qx28’s protective effect (Figure 7). Explants from postnatal day 3 (P3) animals were exposed to gentamicin 100 μM (Figure 7, C and D) or CDDP 8 μM (Figure 7, E and F) alone or in the presence of different concentrations of Qx28 (Figure 7, G and H). Explants were immunostained for the hair cell marker myosin VI and the number of outer hair cells (OHCs) quantified and expressed as the number of OHCs per every 30 inner hair cells. The results from these experiments showed that Qx28 can protect OHCs from gentamicin and CDDP ototoxicity.

### In vitro studies of Qx28’s mechanism of action.

In the decades since the discovery of NF-κB, several paradigms for its function have been established, including key roles in inflammatory and immune responses. NF-κB stimulates immune cell function and can act in a proinflammatory manner by inducing the expression of cytokines, chemokines, and their receptors (54–58). These aspects of NF-κB function seem to be central to the understanding of the overall behavior of this family of transcription factors and provide a foundation for therapeutic interventions based on NF-κB regulation (54–56). However, a wider analysis of NF-κB pathway reveals that most of its activity is cell dependent and dictated by which signaling cascade, canonical or noncanonical, is being activated (55, 56, 58).

Published work has demonstrated a role for NF-κB pathway during ototoxic insult (59), although some controversy exists as to whether its activation results in beneficial or detrimental effects to auditory function (59–65). In AG- and CDDP-induced nephrotoxicity, it is clear that NF-κB activation results in a detrimental effect that leads to kidney failure (11–16, 45). Given this information and given that quinoxaline derivatives can inhibit NF-κB pathway (31), we decided to assess whether Qx28 regulates NF-κB pathway during AG and CDDP exposure.

**Figure 8:**
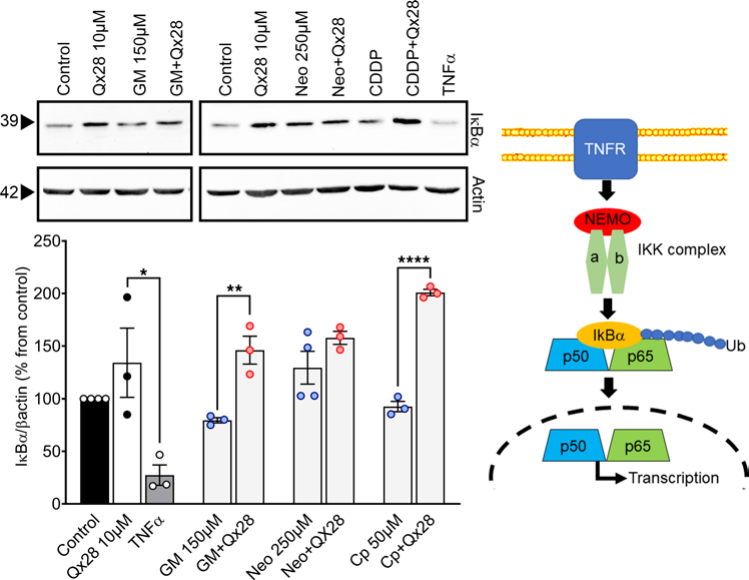
Qx28 modulates NF-κB canonical pathway. Representative immunoblots and quantitative data for IκBα. MEFs were treated for 16 hours with the indicated compounds, then processed for Western blot analysis. Membranes were stripped and reprobed for β-actin as a loading control. TNF-α (10 ng/mL) was used as a positive control for NF-κB canonical pathway activation. Quantification of the bands was performed using ImageJ. Results are expressed as mean ± SEM from 3 independent experiments. Statistical analysis: 2-tailed Student’s *t* test, *P < 0.05, **P < 0.01, ****P < 0.0001 versus the corresponding ototoxin alone. TNF-α treatment was compared with Qx28 10 μM treatment. Cartoon: signaling molecules involved in the NF-κB canonical pathway.

In the canonical pathway, the inhibitor of IκB kinase B (IκKβ) is activated (phosphorylated) by proinflammatory cytokines, such as TNF-α. Active IκKβ can phosphorylate IκBα, targeting it for ubiquitination and proteasomal degradation (56–59). While present, IκBα interacts with NF-κB transcription factors p50 and p65, preventing them from translocating into the nucleus, but upon degradation, p50 and p65 can freely move into the nucleus and induce the expression of proinflammatory genes (Figure 8).

To test whether Qx28 can regulate NF-κB canonical pathway, MEFs were isolated and incubated with TNF-α in the presence or absence of different Qx28 concentrations, followed by the assessment of IκBα abundance by immunoblot studies (Supplemental Figure 1A). Results from these experiments showed that TNF-α alone reduced IκBα abundance (activation of NF-κB pathway) while IκBα values reverted to controls in the presence of Qx28 (inactivation of NF-κB pathway). This result suggests that Qx28 can inhibit the activity of a bona fide NF-κB canonical pathway activator.

The effect of Qx28 in NF-κB canonical pathway was also analyzed in the presence of AGs and CDDP (Figure 8). MEFs were incubated with gentamicin (150 μM), neomycin (250 μM), or CDDP (50 μM) alone or in combination with Qx28 (10 μM) (Figure 8). In the case of MEFs incubated with Qx28 alone, although we observed an increase in IκBα abundance compared with untreated cells, it was not significant. Gentamicin and CDDP incubations resulted in a slight decrease in IκBα abundance compared with controls. However, the coincubation with Qx28 resulted in a significant increase in IκBα abundance (*P* < 0.01 for GM versus GM+Qx28; *P* < 0.0001 for CDDP versus CDDP+Qx28), suggesting that Qx28 is inhibiting NF-κB canonical pathway. The exception was neomycin treatment, in which we did not observe any differences in the abundance of IκBα with or without Qx28, nor when compared with controls, which suggests that the NF-κB canonical pathway may not be involved in neomycin’s mechanism of action.

**Figure 9:**
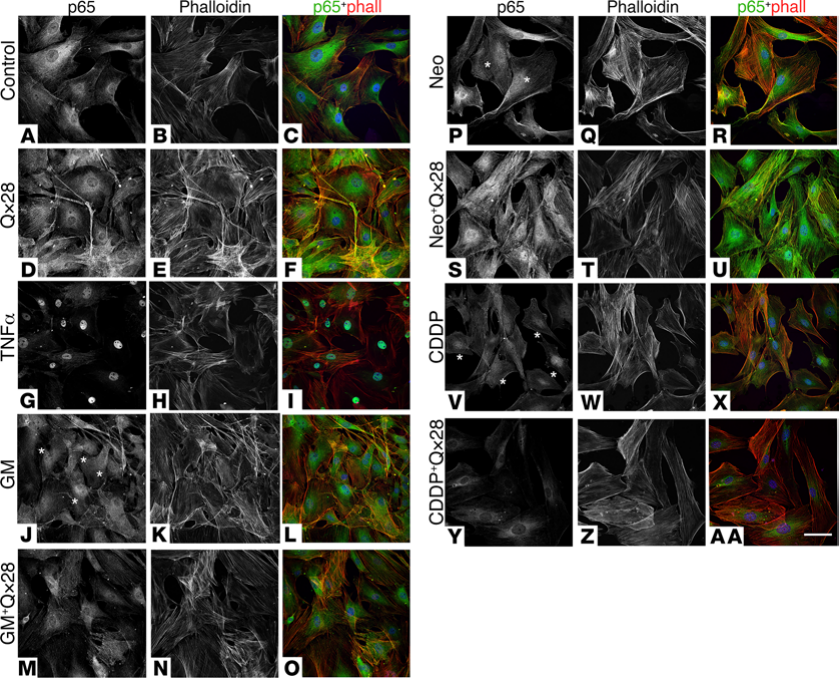
Qx28 modulates p65 nuclear translocation. Representative images of MEFs treated for 16 hours with the indicated compounds: (**A**–**C**) media, (**D**–**F**) Qx28 10 μM, (**G**–**I**) TNF-α 10 ng/mL for 30 minutes (positive control), (**J**–**L**) gentamicin (GM) 150 μM, (**M**–**O**) GM+Qx28, (**P**–**R**) neomycin (Neo) 250 μM, (**S**–**U**) Neo+Qx28, (**V**–**X**) CDDP 50 μM, (**Y**–**AA**) CDDP+Qx28. Cells were immunostained for p65 (green) and counterstained with phalloidin (red). Asterisks denote nuclear translocation. *n* = 2 independent experiments. Scale bar: 55 μm.

Because IκBα degradation leads to p65/p50 translocation into the nucleus and induction of gene expression (54–58), we decided to assess whether Qx28 regulates p65 subcellular localization in MEFs (Figure 9) by confocal microscopy. In the case of controls and MEFs incubated with Qx28 alone (Figure 9, A–F), most p65 was localized in the cytoplasm. It was easy to identify the nucleus by the lack of staining. TNF-α was used as a positive control for p65 nuclear translocation (Figure 9, G–I). Although not as conspicuous as with TNF-α, the incubation with gentamicin and CDDP resulted in translocation of p65 into the nucleus (Figure 9, J–L and V–X, asterisks). This translocation was prevented when the cells were coincubated with Qx28, confirming that Qx28 can inhibit the activation of the NF-κB canonical pathway (Figure 9, M–O and Y–AA). To our surprise, the incubation with neomycin resulted in nuclear translocation of p65 despite the lack of degradation of IκBα observed by Western blot (Figure 8 and Figure 9, P–R). This suggests that neomycin is activating NF-κB through a mechanism that is independent of IκKβ phosphorylation and IκBα degradation as has been previously described for other activators (66). The nuclear localization of p65 was unaffected when MEFs were coincubated with neomycin and Qx28 (Figure 9, S–U), reinforcing the idea of neomycin acting through an alternative NF-κB mechanism. Overall, these results suggest that the 3 ototoxins partially activate NF-κB and that, in the case of gentamicin and CDDP, Qx28 prevents this activation.

Since the effects observed in IκBα and p65 were partial compared with TNF-α, we also assessed the activation state of NF-κB noncanonical pathway (Supplemental Figures 1–3). This pathway can be activated by ligands such as CD40L, lymphotoxin β, and TNF-related weak inducer (TWEAK) and requires the accumulation of the NF-κB–inducing kinase, processing of p100 to p52, and the concomitant translocation of p52 and RelB into the nucleus to induce noncanonical genes (67). We first tested Qx28’s regulation of the noncanonical pathway in MEFs stimulated with TWEAK (Supplemental Figure 1B). The processing of p100 to p52 was used as a proxy for pathway activation. While TWEAK alone resulted in an increase in the p52/p100 ratio (pathway activation) compared with controls, Qx28 was unable to reverse p100 processing, suggesting that Qx28 is not involved in the regulation of the noncanonical pathway.

We next tested whether AGs and CDDP can activate the NF-κB noncanonical pathway. For this purpose, we looked at the increase in the p52/p100 ratio as well as p52 nuclear translocation as indicators of noncanonical NF-κB pathway activation (67). TWEAK was used as a positive control. The results showed that there were no differences between the MEFs treated with the toxins (with or without Qx28) and the MEF control. No increase was observed in the processing of p100 (Supplemental Figure 2), or in the nuclear translocation of p52 (Supplemental Figure 3), suggesting that the noncanonical pathway is not involved in AG’s and CDDP’s deleterious effect.

### In vivo studies of Qx28’s mechanism of action.

**Figure 10:**
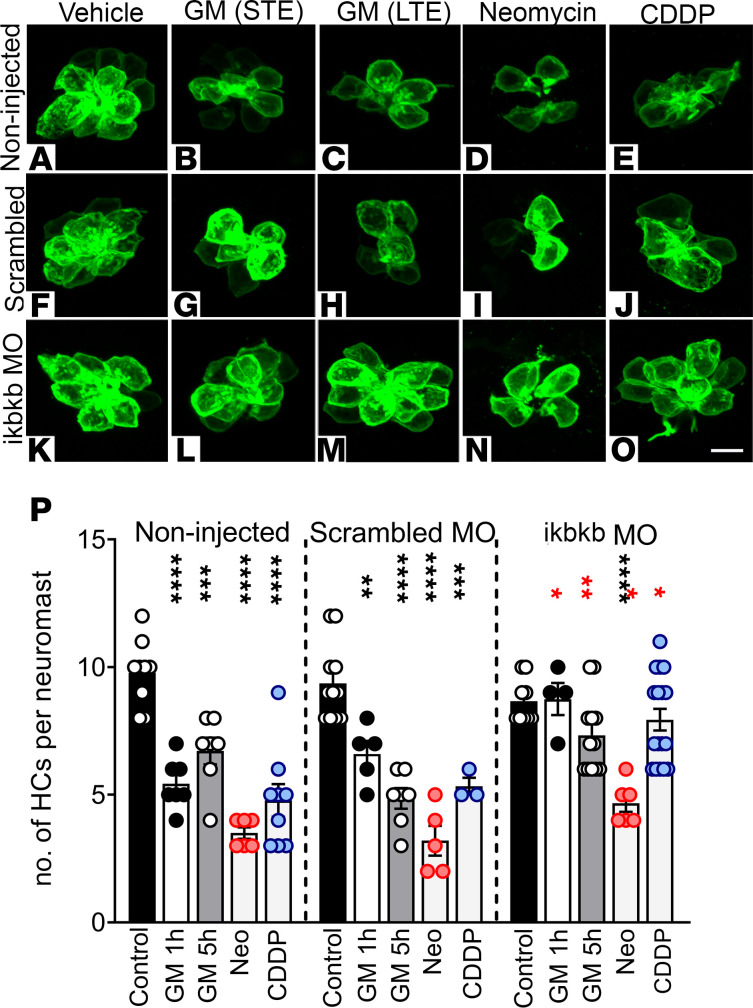
Reduction of IκKβ expression protects against aminoglycoside- and CDDP-induced ototoxicity. (**A**–**O**) *Tg(brn3c:GFP)* zebrafish eggs were noninjected (**A**–**E**) or injected with a suboptimal dose (2 ng) of scrambled (**F**–**J**) or *ikbkb* morpholinos (MO) (61) (**K**–**O**). At 3 dpf animals were incubated with gentamicin (GM) 100 μM STE (**B**, **G**, and **L**) or LTE (**C**, **H**, and **M**), neomycin (Neo) 200 μM (**D**, **I**, and **N**), or CDDP 400 μM (**E**, **J**, and **O**). Animals were fixed and immunostained for GFP. The number of hair cells per neuromast was quantified (**P**). Results are expressed as mean ± SEM. Statistical analysis: 1-way ANOVA with correction for Dunnett’s multiple comparisons test. **P < 0.01; ***P < 0.001; ****P < 0.0001 versus the corresponding control within the group (black asterisks). Two-tailed Student’s *t* test. *P < 0.05; **P < 0.01 versus identical treatment in scrambled animals (red asterisks). Number of neuromasts inspected: control = 8 (noninjected), 12 (scrambled MO), 10 (IκKβ MO); GM-STE = 7 (noninjected), 5 (scrambled), 4 (*ikbkb* MO); GM-LTE = 7 (noninjected, scrambled), 12 (*ikbkb* MO); Neo = 6 (noninjected, *ikbkb* MO), 5 (scrambled); CDDP = 9 (noninjected), 3 (scrambled), 12 (*ikbkb* MO). Scale bar: 6 μm.

We then decided to test whether the NF-κB pathway is also activated in vivo employing 2 approaches: (a) *ikbkb* knockdown in zebrafish and (b) activation of the NF-κB pathway in the reporter zebrafish line *Tg*(*NFKB:EGFP)* (35). Since it has already been shown that IκKβ is a key target of quinoxaline derivatives (31), we knocked down its expression in zebrafish by the use of *ikbkb* morpholinos, followed by incubation of the zebrafish morphants with the different ototoxins (Figure 10). If Qx28 protects hair cells by inhibiting IκKβ activity, then *ikbkb* morphants will be protected from ototoxin exposure, even in the absence of Qx28. Eggs from the *Tg(brn3c:GFP)* line were injected with *ikbkb* or scrambled morpholinos at a suboptimal dose of 2 ng (68), then incubated with or without the different toxins at 3 dpf. Under basal conditions (no ototoxin), scrambled and *ikbkb* zebrafish morphants did not show any differences in the number of hair cells per neuromast compared with noninjected animals (Figure 10, A, F, K, and P), suggesting that, per se, the morpholinos are not toxic to the hair cells. The exposure of scrambled morphants to gentamicin (acute and long-term exposure), neomycin, or CDDP resulted in a similar amount of hair cell loss as in noninjected animals exposed to the same insults (Figure 10, B–E, G–J, and P). Conversely, *ikbkb* morphants were able to retain their hair cells when exposed to gentamicin or CDDP, suggesting that this is, indeed, the pathway activated by these 2 ototoxins (Figure 10, L, M, O, and P). In neomycin exposure experiments, *ikbkb* when knocked down did not show any protective effect, reinforcing the notion that neomycin is not acting through the NF-κB canonical pathway (Figure 10, N and P).

To confirm the absence of general toxicity after morpholino administration and *ikbkb* knockdown, we assessed the gross morphology of 3 dpf zebrafish morphants and performed reverse transcriptase PCR (RT-PCR), respectively (Supplemental Figure 4). Morphological assessment of the *ikbkb* morphants did not show any general abnormality compared to scrambled (Supplemental Figure 4B) or noninjected animals (data not shown), suggesting *ikbkb* knockdown did not result in any toxic effect. Moreover, RT-PCR showed the reduction of the *ikbkb* transcript in the *ikbkb* morphants (Supplemental Figure 4A).

**Figure 11:**
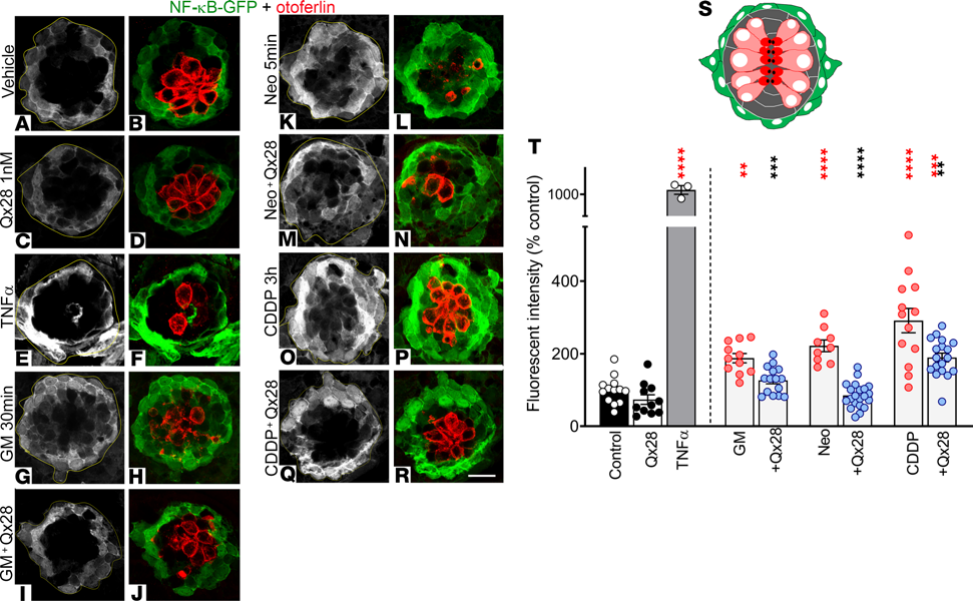
Qx28 protects hair cells by NF-κB pathway inhibition in vivo. Five dpf *Tg*(*NFKB:EGFP)* were treated with E3 water (**A** and **B**), Qx28 1 nM (**C** and **D**), TNF-α 20 ng/mL 30 minutes (positive control) (**E** and **F**), gentamicin (GM) 100 μM 30 minutes without (**G** and **H**) or with (**I** and **J**) Qx28, neomycin (Neo) 200 μM 5 minutes without (**K** and **L**) or with (**M** and **N**) Qx28, and CDDP 400 μM 2 hours without (**O** and **P**) or with (**Q** and **R**) Qx28. Animals were transferred to E3 water for 2 hours and immunostained for GFP (green) and otoferlin (red). The green fluorescence intensity was quantified using ImageJ and expressed as percentage from control (**T**). (**S**) Cartoon depicting a neuromast (top view): hair cells are in red, supporting cells are in gray, and mantle cells are in green. Results are expressed as mean ± SEM. Statistical analysis: 2-tailed Student’s *t* test, **P < 0.01, ***P < 0.001, ****P < 0.0001 versus ototoxin alone (black asterisks). One-way ANOVA with correction for Dunnett’s multiple comparisons test. **P < 0.01; ***P < 0.001; ****P < 0.0001 versus vehicle (red asterisks). Number of neuromasts quantified: *n* = 13 (control, CDDP), 11 (Qx28, GM), 3 (TNF-α), 15 (GM+Qx28), 9 (Neo), 20 (Neo+Qx28), 18 (CDDP+Qx28). Scale bar: 9 μm.

Although the experiments performed with the *ikbkb* morphants implicate NF-κB canonical pathway as one of the main pathways activated by gentamicin and CDDP, they do not provide any information regarding the in vivo role of Qx28 in NF-κB pathway regulation. To accomplish this, we took advantage of the NF-κB reporter zebrafish line, *Tg*(*NFKB:EGFP)*, which expresses GFP under the control of an NF-κB promoter sequence (35). To assess NF-κB activation in vivo, zebrafish were pretreated with a Qx28 dose that confers protection (Figure 3) without any evidence of MET channel blockage (Figure 5) or AG uptake impairment (Figure 6). After 1-hour pretreatment, animals were coincubated with the ototoxin and Qx28 for a short period (to reduce hair cell loss), let to recover for 2 hours to allow GFP synthesis, and then fixed and immunostained for GFP and the hair cell marker otoferlin (Figure 11). Under these experimental conditions, we only observed GFP expression in the neuromast supporting cells, more likely the mantle cells (Figure 11S). Neuromast hair cells were completely devoid of GFP immunostaining. Basal GFP expression levels were present in control animals, measured as total fluorescence intensity (Figure 11, A and B). These levels were unchanged when larvae were exposed to 1 nM (Figure 11, C and D, and T) or 100 μM of Qx28 (data not shown). However, when animals were incubated with the different ototoxins, we observed a significant increase in GFP fluorescence intensity compared with vehicle or Qx28 alone (Figure 11, G, H, K, L, O, P, and T). Coincubation of Qx28 with the ototoxin resulted in a significant decrease in GFP levels (Figure 11, I, J, M, N, and T), although in the case of CDDP, GFP fluorescence never reached control values (Figure 11, O–R and T). This last result agrees with the partial protection conferred by Qx28 against CDDP and suggests that NF-κB is not the only deleterious pathway activated by it. Overall, these results suggest Qx28 interferes with ototoxin’s ability to kill hair cells by modulating the NF-κB pathway in supporting cells in vivo.

## Discussion

Hearing loss is a major health concern, affecting over 460 million people worldwide (1). The detrimental effects of hearing loss go well beyond hearing alone; they limit people’s quality of life, restrict employment and job performance, affect recreational and social activities, compromise safety, and, ultimately, can lead to depression and social isolation (69). Thus, identifying novel therapeutic interventions that will stop or slow the progression of this disease is imperative.

AGs are broad-spectrum antibiotics widely used to treat serious Gram-negative bacterial infections and although they are extremely effective, they carry the risk of adverse side effects, including irreversible damage to the inner ear (12, 14, 17–21). Several studies have pointed to the importance of quinoxaline derivative structures as a treatment for infectious diseases, cancer, tinnitus, as well as different types of neurodegenerative disorders (30, 57, 70–76). Our previous work demonstrated the partial protection of quinoxaline against AG-induced ototoxicity (27). Based on that information we undertook the task of designing and synthesizing quinoxaline analogs to improve their efficacy and potency for protection against antibiotic ototoxicity. These new chemical derivatives (Qx2 to Qx70) were systematically screened in a zebrafish model for AG ototoxicity. We found many derivatives that were protective against neomycin or gentamicin, but only a few (approximately 14) performed better than quinoxaline. Within those few compounds, Qx28 (quinoxaline-5-carboxylic acid) was the only derivative that protected hair cells against both neomycin and gentamicin ototoxicity. An initial comparative analysis of the quinoxaline derivatives’ structure-activity relationship identified the carboxylic acid in position 5 in Qx28 as an important chemical group for otoprotection against AGs. Further characterization in zebrafish experiments confirmed that Qx28 was better than quinoxaline for protection against AG-induced hearing loss: (a) Qx28 was more potent, and we were able to protect at concentrations that were approximately 100,000 lower than quinoxaline; and (b) Qx28 showed higher efficacy, and Qx28 treatment resulted in less hair cell loss compared with quinoxaline.

One feature that makes hair cells vulnerable to AG toxicity is their ability to incorporate and accumulate AGs (50). While AGs can be easily eliminated from the bloodstream, hair cells from the inner ear can actively take up and accumulate them, resulting in severe ototoxicity (77). Moreover, only functional hair cells can incorporate AGs, suggesting that the MET machinery is directly or indirectly involved in this process (30, 51, 78–80). Since it has been shown that many otoprotectants, including some quinoxaline derivatives, exert their effect by blocking hair cell MET channel activity (81), we decided to evaluate whether that was the case for Qx28. FM1-43 dye uptake experiments and microphonic potential measurements showed that only at high doses Qx28 acts as a partial MET blocker. Doses lower than 100 μM were otoprotective despite minimal or no effect on MET channel activity, suggesting that Qx28 is acting through an alternative mechanism and that the inhibition of the MET channels is, more likely, a side effect of Qx28 treatment at high concentrations. The experiments with the AGTRs not only confirmed this but also showed that the toxin has access to the intracellular milieu even in the presence of Qx28.

These results together with the fact that Qx28 does not interfere with antibiotic efficacy strongly point to an inhibitory effect of Qx28 into the ototoxin’s mechanism of action. Furthermore, they provide a proof of principle for the development of more efficacious and potent compounds while preserving the desired characteristics of the original quinoxaline molecule (i.e., preservation of both MET channel activity and AG antibacterial activity, ref. 27).

The observation that Qx28 protects zebrafish hair cells from AG ototoxicity prompted us to test whether Qx28 can confer protection in a more relevant model for hair cell damage, such as mouse cochlear explants. Experiments with gentamicin confirmed that similar to zebrafish, Qx28 protected mammalian hair cells from AG damage.

When studying NF-κB pathway activation we found that while incubation of MEFs with gentamicin resulted in the activation of NF-κB canonical pathway, neomycin induced NF-κB via an alternative mechanism that was independent of IκKβ (Qx28 did not have any effect) or the noncanonical pathway (there was no processing of p100) (67). Conversely, when studying the involvement of NF-κB pathway in zebrafish, we observed that both AGs increased NF-κB activity in the reporter zebrafish line *Tg*(*NFKB:EGFP)* and that GFP values were restored to basal levels when animals were cotreated with Qx28, suggesting that both AGs activate the NF-κB canonical pathway. Moreover, *ikbkb* morphants were resistant to AG ototoxicity. However, while *ikbkb* morphants were completely refractive to gentamicin’s deleterious effect, neomycin protection was only partial. This last result suggests that although neomycin mediates the activation of the NF-κB canonical pathway in fish, this is not its main mechanism of action for hair cell loss. The fact that Qx28 cotreatment, which inhibits neomycin-induced NF-κB activation, and *ikbkb* knockdown, which blocks NF-κB canonical pathway activation, are not sufficient to confer full protection strongly supports this idea of an alternative mechanism for neomycin ototoxicity. In summary, from the NF-κB pathway analysis studies, we conclude, that for gentamicin: (a) the main mechanism of action is through NF-κB canonical pathway activation, (b) this mechanism is conserved between mouse and fish, and (c) the treatment with Qx28 results in otoprotection due to a reduction in NF-κB activity through, more likely, IκKβ inhibition. In the case of neomycin we can conclude that: (a) its main mechanism of action associated with hair cell death is not through the NF-κB pathway, (b) the pathways activated by neomycin seem to differ between species, and (c) since NF-κB is not neomycin’s main mechanism of action, Qx28 confers partial protection at doses that do not block MET channels.

Notably, our findings showed that the initial induction and modulation of the NF-κB pathway occurred in the neuromast supporting cells, suggesting a critical function for these cells during AG-induced ototoxicity. Intercellular communication in the mammalian cochlea, due to AG ototoxicity, has recently been described by Breglio et al. (82). That work demonstrated that the release of heat shock 70 kDa protein–containing exosomes from supporting cells can improve hair cell protection against neomycin. In our case, the Qx28-mediated reduction in NF-κB pathway activation helped prevent hair cell death, suggesting possible deleterious factors being released by the affected supporting cells.

Finally, given that quinoxaline conferred protection against CDDP in zebrafish, we decided to test whether Qx28 was also protective against CDDP ototoxicity. Such studies showed that Qx28 not only preserved quinoxaline’s properties but also performed better, with higher potency and similar effectiveness. Both fish and mouse experiments demonstrated the potential of Qx28 as a therapeutic compound against CDDP’s ototoxicity. Moreover, CDDP-mediated NF-κB pathway activation was blocked by Qx28 in the 2 species, suggesting this is the main common mechanism of action through which Qx28 protects hair cells.

The mechanisms of ototoxicity identified in recent years by using different types of experimental models (6–8, 21, 26–28, 36–38) have increased our knowledge for the development of novel therapies against specific molecular targets involved in hair cell death. Because susceptibility to AG- and CDDP-induced hearing loss varies, alternative and novel strategies are needed to identify new compounds that will provide additional treatments for protection against drug-induced hearing loss. With this idea in mind, we have identified Qx28 as the best compound against AG- and CDDP-induced ototoxicity. Unlike some agents currently in preclinical or clinical trial phases, which act at later stages of reactive oxygen species (ROS) generation, when irreversible oxidative damage to the auditory hair cells has already happened (6, 22), Qx28 acts upstream of ROS production, preventing activation of the NF-κB pathway (31), thus protecting hair cells from the cascade of apoptotic events triggered by it. Taken together, our results suggest Qx28 can protect hair cells from AG- and CDDP-induced hair cell loss by inhibiting NF-κB activation.

Since Qx28’s chemical properties follow Lipinski’s rule of five (83), we can predict with confidence that, if systemically administered, Qx28 will reach the inner ear compartment. However, more studies are warranted regarding Qx28’s capability to cross the BLB. Additionally, future studies will assess whether Qx28 can alleviate iatrogenically induced hearing loss in more relevant animal models. Because noise exposure results in NF-κB pathway activation (59), Qx28’s potential for the treatment of noise damage will also be explored.

## Methods

### Quinoxaline derivative synthesis

All reactions were carried out in an oven-dried round-bottom flask with magnetic stirring under an open atmosphere. Reagents were purchased from MilliporeSigma, Acros, or Alfa Aesar. Before use, solvents were treated with 4 Å molecular sieves. The purification of the reaction products was carried out by chromatography using CHEM-LAB silica gel (230–400 mesh). The purity of the quinoxaline derivatives was tested by HPLC, and only compounds with more than 95% purity were used for the experiments. ^1^H NMR and ^13^C NMR spectra were recorded with tetramethylsilane as an internal standard at ambient temperature on a Bruker 400 Avance III HD for ^1^H NMR and a 100 MHz for ^13^C NMR. Chemical shifts were reported in parts per million and coupling constants in hertz. Splitting patterns were designated as a singlet, broad singlet, doublet, triplet, doublet of a doublet, or triplet of a triplet. Splitting patterns that could not be interpreted or easily visualized were designated as multiple. Spectroscopic data of all the compounds matched with the previously reported data.

In general, a stoichiometric quantity of substituted aromatic diamine was condensed in the presence of a strong acidic condition, which undergoes oxidation to furnish the quinoxaline derivatives. The progress of the reaction was monitored by thin layer chromatography. Once the complete consumption of the starting material was confirmed, the reaction mixture was quenched with a saturated solution of sodium bicarbonate, extracted twice with ethyl acetate, and dried over anhydrous sodium sulfate. The combined organic layer was then evaporated under vacuum, and the resulting crude was purified by column chromatography on silica gel with an increasing concentration of ethyl acetate in hexane to afford the desired compound.

Some of the quinoxaline derivatives were purchased from MilliporeSigma, Acros, or Alfa Aesar (Supplemental Table 1).

Stock solutions were prepared in DMSO at 50–100 mM. The working solutions never contained more than 1% DMSO.

### Animals

Zebrafish (*Danio rerio*) experimental larvae were obtained by pair mating of adult fish maintained at Creighton University by standard methods approved by the Institutional Animal Care and Use Committee. We used TuAB WT fish, *Tg(brn3c:GFP)*, *Tg*(*NFKB:EGFP)*, and *orbiter* zebrafish lines (34, 35, 52, 53). The *Tg(brn3c:GFP)* line expresses a membrane-bound GFP in hair cells. The *Tg*(*NFKB:EGFP)* line expresses an inducible GFP under NF-κB control. The *orbiter* line carries a mutation in *Pcdh15a* resulting in tip link disruption. Experimental fish were maintained at 28.5°C in E3 media (5 mM NaCl, 0.17 mM KCl, 0.33 mM CaCl_2_ and 0.33 nM MgSO_4_, pH 7.2). Animals were cryoanesthetized after drug treatment and prior to fixation. The neuromasts inspected were part of the cranial system and included the otic, middle, and opercular neuromasts.

FVB breeding mice were housed in the Animal Resource Facility at Creighton University with a 12-hour light/12-hour dark cycle and free access to food and water. Experimental animals were used at P3. Their inner ear was microdissected, and the organ of Corti was isolated and used for cochlear explant experiments.

### Zebrafish screening and Qx28 studies

Initial screening of the quinoxaline derivatives was performed at 300 μM since this was the concentration for which quinoxaline showed the best results against AG ototoxicity (27). Those derivatives showing the same level of protection as quinoxaline were further characterized at lower doses until they did not show any significant difference compared with neomycin or gentamicin alone. The lower doses were chosen to cover a 10,000 concentration range (300 μM to 1 μM and 10 nM, Supplemental Table 1). For the screenings, 5 dpf *Tg(brn3c:GFP)* larvae were preincubated with one of the quinoxaline analogs for 1 hour followed by coincubation with the ototoxin. For gentamicin LTE, animals were exposed to 100 μM of gentamicin (MilliporeSigma G1914) for 1 hour, transferred to E3 water for 5 hours, and fixed in 4% paraformaldehyde (PFA) overnight (27). For neomycin (MilliporeSigma N1876), animals were exposed to 200 μM for 30 minutes, transferred to E3 water for 1 hour, and then fixed. Neuromast hair cells were immunolabeled with anti-otoferlin (HCS-1, DSHB) and anti-GFP (NB100-1614, Novus Biologicals). These 2 markers were used to detect and count neuromast hair cells since we previously noticed that the incubation with the toxins can affect protein expression in a way that complicates their detection under a fluorescence microscope (27). Using both GFP and otoferlin for hair cell count reduced the chances of missing some of the hair cells after the treatment. Otic, middle, and opercular neuromasts were identified, and hair cells were manually counted using a Zeiss AxioSkop 2 fluorescence microscope with a 40× oil objective.

For Qx28 characterization, 5 dpf *Tg(brn3c:GFP)* larvae were preincubated with Qx28 or quinoxaline (1 nM to 300 μM) and then coincubated with gentamicin (100 μM) for 1 hour, neomycin (200 μM) for 30 minutes, or CDDP (400 μM, MilliporeSigma, 479306) for 6 hours. Animals were then transferred to E3 water for 1 hour before processing for immunohistochemistry (27). Control animals were incubated in E3 fish water containing 0.1% DMSO. Hair cells were quantified as described above for the library screening, and images were taken employing a Zeiss LSM 800 system with Airyscan function employing a 63× oil objective.

### FM1-43 uptake

FM1-43 dye uptake was performed as previously described (27). Five dpf WT zebrafish were incubated for 1 hour with Qx28 and then exposed for 20 seconds to 3 μM of FM1-43 in the presence of Qx28. Animals were fixed and counterstained with Alexa Fluor 488–conjugated phalloidin. Images were taken employing a Zeiss LSM 700 employing a 63× oil objective. The fluorescence incorporated per neuromast was quantified using ImageJ (NIH) (27).

### Microphonic potentials

Microphonic responses were recorded from neuromasts using the technique described elsewhere (27). In brief, zebrafish were embedded in agarose and mounted in the experimental chamber. A glass paddle with a tip diameter of 3 μm was positioned 5 μm near a neuromast. The displacement of the paddle, driven by a piezoelectric actuator (Burleigh Driver/Amplifier, PZ-150M), was calibrated by a photodiode-based system (84). The movement of the paddle was coupled to the hair bundle displacement through the fluid. Sinusoidal bursts with frequencies of 100 or 200 Hz were used. The displacement of the probe was set at approximately 3 μm, sufficient to generate a maximum (saturated) microphonic response.

Patch electrodes were used to record microphonic potentials. The recording electrode had open tip resistances of approximately 5–7 MΩ when filled with standard fish saline solution (29.6 mM NaCl, 2.7 mM KCl, and 1.8 mM CaCl_2_ with pH of 7.2). The microphonic responses (filtered at 1  kHz) were amplified using an Axopatch 200B amplifier and acquired using pClamp 10 (Axon Instruments) running on an IBM-compatible computer with a 16-bit A/D converter (Digidata 1442). Twenty averages were preset for each recording. Data were analyzed using Clampfit in the pClamp software package and Igor Pro (WaveMetrics, Inc.).

### AGTR uptake experiments

Gentamicin and neomycin were conjugated to Texas Red as previously described (27, 60, 62) and diluted in E3 media to the final working concentrations of 50 μM for gentamicin and 100 μM for neomycin. These AG concentrations correspond to half the doses used in our otoprotection experiments and were chosen to reduce hair cell loss. Animals were preincubated with Qx28 1 nM, 1 μM, or 100 μM and then coincubated with the AGTR for 45 seconds, 15 minutes, or 30 minutes. Zebrafish incubated in E3 media containing Texas Red alone were used as controls.

### Preparation of cochlear explants and drug exposure

Cochleae were removed from P3 FVB mice and placed in precooled sterile HBSS (MilliporeSigma, H6648). The organ of Corti was dissected and maintained in culture medium (DMEM/F12 containing 1% FBS, 2% B-27 supplement, 1% N-2 supplement, and 50 μg/mL ampicillin). After overnight incubation at 37°C in 5% CO_2_, the medium was replaced with growth media with or without Qx28 (10 nM–1 mM). After 1-hour incubation, fresh media containing 100 μM gentamicin (MilliporeSigma, G1397) or 8 μM CDDP (Accord, NDC16729-288-11) was added (with or without Qx28). Explants were incubated for 24 hours with the gentamicin or for 48 hours with CDDP, PFA fixed for 10 minutes, and then processed for immunohistochemistry. The primary antibody incubation was performed overnight at 4°C, followed by secondary antibody (donkey anti-rabbit Alexa Fluor 594, catalog A32754, Thermo Fisher Scientific) incubation for 2 hours at room temperature. Rabbit anti–myosin VI (catalog 25-6791, Proteus Bioscience) was used at a 1:400 dilution. Confocal imaging was performed using a Zeiss LSM 700 confocal system with a 40× oil objective; 200 μm regions from the middle turn were photographed, and the number of intact OHCs in each 30 inner hair cells was counted.

### AG efficacy studies

AG efficacy tests (27) were performed to assess Qx28 interference with AG antibiotic activity. Briefly, 6 mm filter discs were soaked overnight in PBS, Qx28 100 μM, gentamicin, or neomycin 10 μg/mL or in combination with AG and Qx28. *E*. *coli* (strain ATCC25922) was plated in agar plates, and the filter discs were leaned on top of them. Plates were incubated overnight and imaged, and the inhibitory area was calculated for each condition and expressed as square millimeters.

### MEF experiments

MEFs were prepared according to Berthet et al. (85) from embryonic day 13–14 FVB mice. Fibroblasts were plated in 6-well plates for Western blot studies or in slides for immunofluorescence studies and used when they reached 70%–80% confluence.

#### TNF-α and TWEAK stimulation.

MEFs were grown overnight in DMEM with 1% FCS before TNF-α (Cell Signaling Technology, catalog 5178) or TWEAK (PeproTech, catalog 10770-808) stimulation. On the day of the experiment, cells were preincubated for 1 hour with Qx28 1 nM to 10 μM and then coincubated with murine TNF-α (mTNF-α) (10 ng/mL) for 30 minutes or human TWEAK (hTWEAK) (20 ng/mL) for 4 hours. Cells were immediately harvested for immunoblot studies.

#### AG and CDDP treatments.

AG and CDDP incubations were done overnight in DMEM with 1% FCS in the presence or absence of Qx28 10 μM and gentamicin (150 μM), neomycin (250 μM), or CDDP (50 μM). Immunoblots were performed as previously described (53). mTNF-α and hTWEAK were included as controls for the activation of the canonical and noncanonical NF-κB pathways, respectively (67).

#### Primary antibodies.

Anti-IκBα (1:500, Cell Signaling Technology catalog 4812), anti–NF-κB p100/p52 (1:500, Cell Signaling Technology catalog 4882), and anti–β-actin (1:2000, MilliporeSigma catalog A5441) were used. Specific bands were detected using the iBright FL1000 system (Thermo Fisher Scientific) and quantified with ImageJ. Results were expressed as a percentage from controls.

#### Immunofluorescence studies.

MEFs were incubated with the different drug combinations as described above and fixed for 15 minutes with 4% PFA. After 5-minute permeabilization in PBS Triton X-100 0.1%, cells were stained with anti–NF-κB p65 (1:500, Cell Signaling Technology, catalog 8242) or anti–NF-κB p100/p52 and counterstained with phalloidin and DAPI.

### Generation of morphants and Qx28 studies

*ikbkb* morphants and scrambled morphants were generated according to Correa et al. (68), resulting in suboptimal IκKβ protein knockdown. Morphants were used at 3 dpf for the ototoxicity studies, fixed, and immunostained with anti-GFP, and the neuromast hair cells were imaged under an LSM 700 confocal microscope.The *ikbkb* primers were AAGTTTCAGGAAGTAGAGAAACTC and AGAGAGTGACGTTGCCAAATC. The *actb* primers were CAGACATCAGGGAGTGATGG and CAACACGGAGCTCATTGTAGA.

### Qx28 studies with the *Tg(NFKB:EGFP)* reporter zebrafish line

The reporter line *Tg*(*NFKB:EGFP)* was obtained from The Zebrafish Information Network and used for in vivo studies of NF-κB regulation. Five dpf animals were preincubated for 1 hour with vehicle (DMSO 0.1% in E3 water) or Qx28 1 nM followed by coincubation with gentamicin 100 μM for 30 minutes, neomycin 250 μM for 5 minutes, or CDDP 400 μM for 2 hours. The incubation times were chosen to avoid hair cell death due to the ototoxin. Animals were transferred to E3 water for 2 hours to allow GFP synthesis and then fixed and prepared for confocal microscopy studies.

### Confocal imaging

For the screening of the quinoxaline library, neuromast hair cell counts were performed manually, employing a Zeiss AxioSkop 2 fluorescence microscope with a 40× oil objective.

Confocal imaging was performed using a Zeiss LSM 700 confocal laser scanning image system with a 40× oil objective or a Zeiss LSM 800 system (Airyscan function) with a 63× oil objective (Carl Zeiss). Images were captured at room temperature with automatically set sectioning. The acquired images were processed with ZEN black edition software. *Z*-stack images are presented as flat *Z*-projections. Only linear adjustments were made to brightness and contrast, and the final figures were assembled using Photoshop and Illustrator software (Adobe).

### Statistics

Two-tailed Student’s *t* test or 1-way ANOVA followed by Dunnett’s multiple comparisons test were performed using GraphPad Prism version 8.2.0 software. *P* values less than 0.05 were considered significant. Results are expressed as mean ± SEM. For the experiments performed with fish, 5–8 fish were used per experiment. For the explant experiments, 2–3 P3 explants were inspected per treatment. Western blots were performed 3 times employing 3 different biological replicates. The representative images shown for the MEF immunocytochemistry experiments correspond to 2 independent biological replicates.

### Study approval

All the experiments performed with live animals were in accordance with the guidelines and regulations approved by the Institutional Animal Care and Use Committee at Creighton University.

## Author contributions

ZX, SH, MZ, and JZ conceived the experiments. ZX, SH, WH, and MZ performed experiments. HL and DZH performed the microphonic experiments. ZX, SH, and MZ prepared tables and figures. ZX, MZ, and JZ performed data analyses. MZ wrote the manuscript.

## Supplementary Material

Supplemental data

## Version 1 01/21/2021

In-Press Preview

## Version 2 03/08/2021

Electronic publication
