# Supplementary material for: Characterization of quinoxaline derivatives for protection against iatrogenically induced hearing loss
Source: JCI Insight. 2021 Mar 8;6(5):e141561. doi: 10.1172/jci.insight.141561 (PMC8021103; doi:10.1172/jci.insight.141561)
Supplement: Supplemental data [file jciinsight-6-141561-s213.pdf]

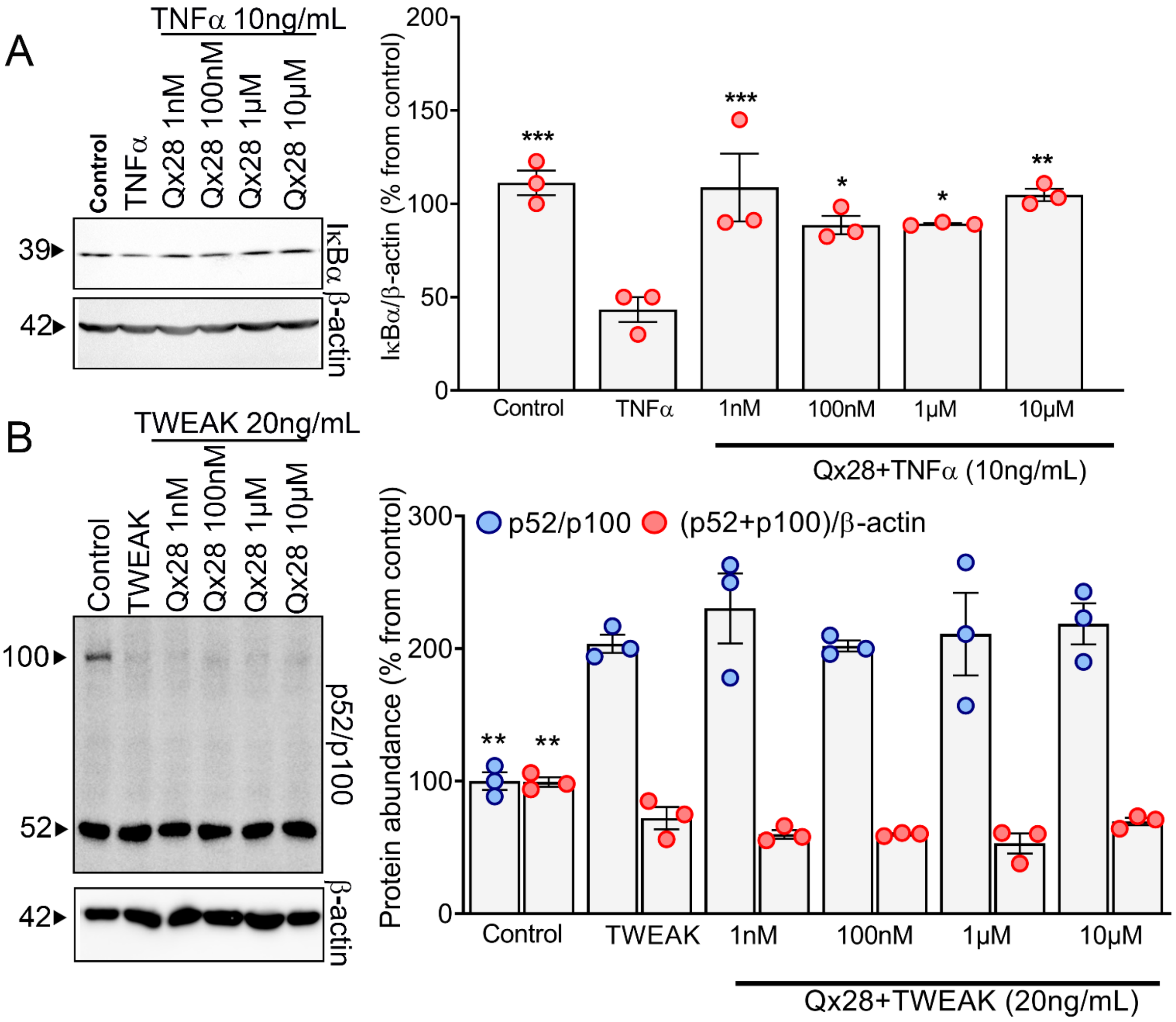

Figure S1. *Qx28 inhibits TNFα-mediated activation of the NF-κB canonical pathway but has no effect on TWEAK-mediated activation of the NF-κB non-canonical pathway.* **A:** Representative immunoblot and quantitative data for IkBα. MEFs were grown overnight in 1% FCS/DMEM, pre-incubated for 1-hour with media alone or Qx28, and then co-incubated for 30-min with TNFα (10ng/mL). **B:** Representative immunoblot and quantitative data for p52:p100 ratio and total p52/p100. MEFs were grown overnight in

1% FCS/DMEM, pre-incubated for 1-hour with media alone or Qx28, and then co-incubated for 4-hrs. with TWEAK (20ng/mL).

Membranes were stripped and re-probed for β-actin as a loading control. Quantification of the bands was performed using ImageJ (NIH). Results are expressed as mean +/- SEM from 3 independent experiments. Statistical analysis: One-way ANOVA with correction for Dunnett's multiple comparisons test. *$P<0.05$; **$P<0.01$; ***$P<0.001$ versus TNFα or TWEAK alone.

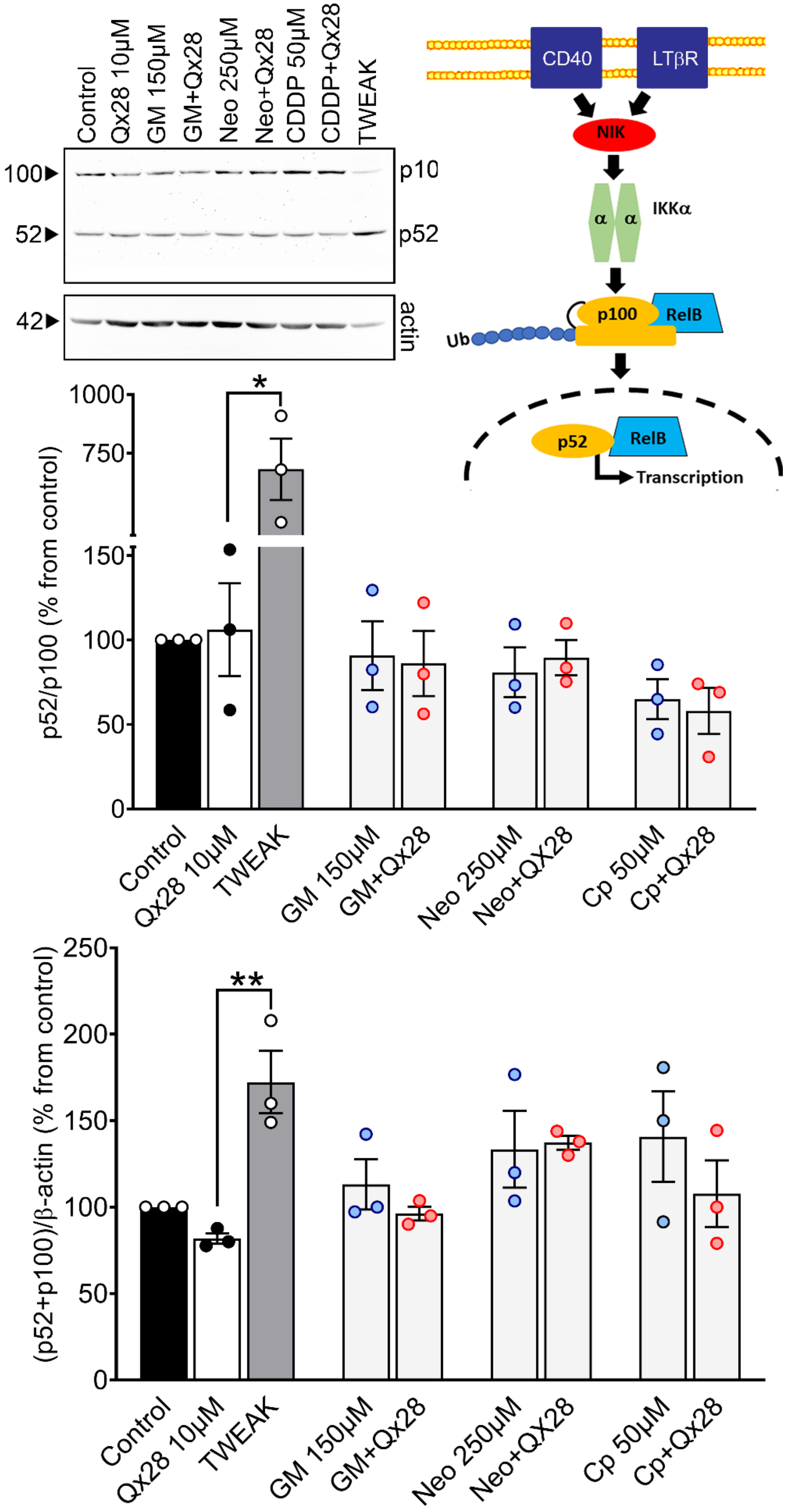

Figure S2. *Aminoglycosides and CDDP are not involved in the activation of the NF-κB*

non-canonical pathway. Representative immunoblot and quantitative data for p52:p100 ratio and total p52+p100. MEFs were incubated for 16-hours with the corresponding treatment in 1% FCS media and then processed for western blot analysis. Membranes were stripped and re-probed for β-actin as a loading control. TWEAK (20ng/mL) was used as a positive control for NF-κB non-canonical pathway activation. Quantification of the bands was performed using ImageJ (NIH). Results are expressed as mean +/- SEM from 3 independent experiments. Statistical analysis: Two-tailed Student's t test versus the corresponding ototoxin. *$P<0.05$, **$P<0.01$ Qx28 10μM versus TWEAK. Cartoon depicts some of the signaling molecules involved in the NF-κB non-canonical pathway.

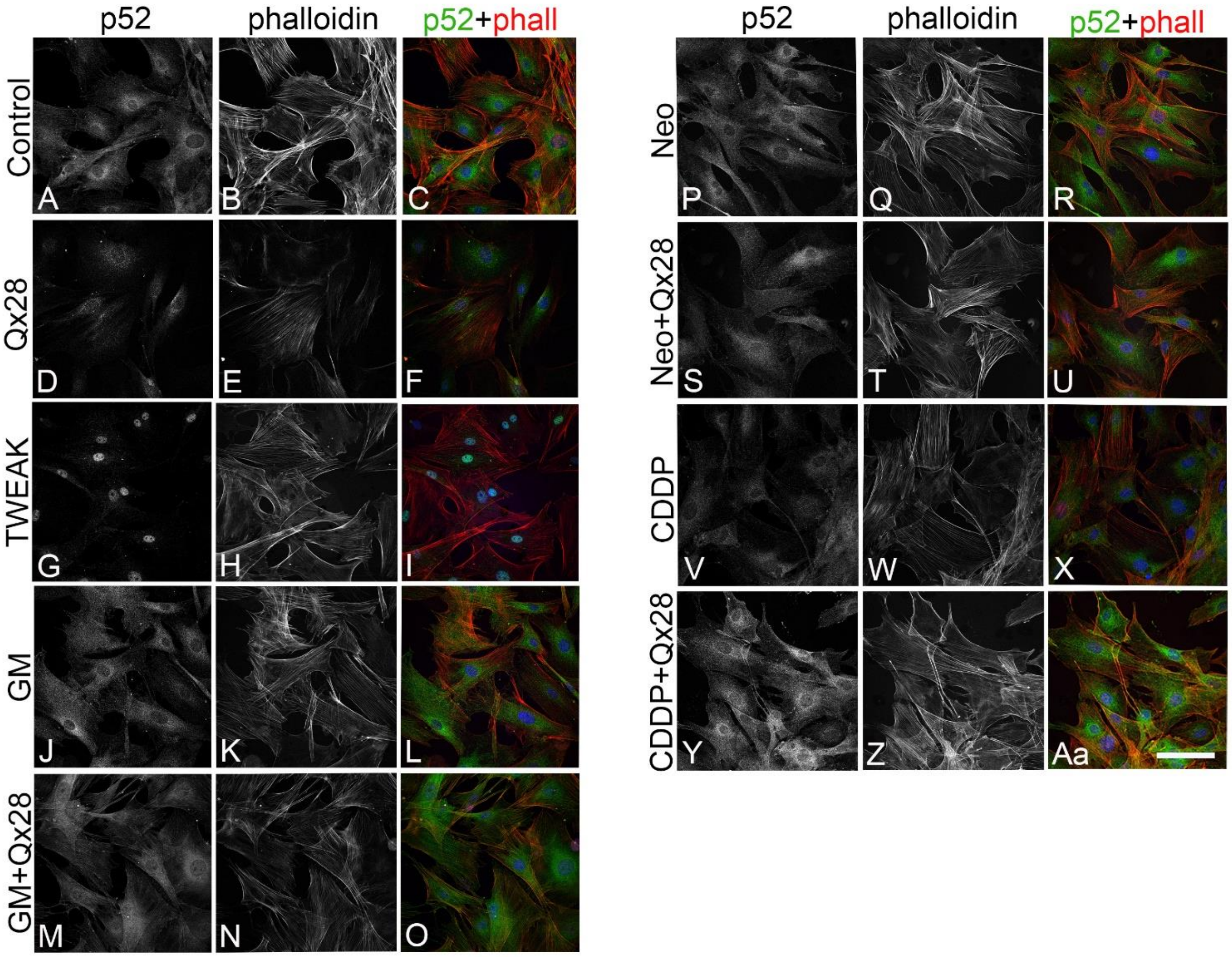

Figure S3. *Aminoglycosides and CDDP have no effect on p52 subcellular localization*. Representative images of MEFs incubated for 16-hours with 1% FCS media and the indicated treatment. **A-C:** media; **D-F:** Qx28 10μM; **G-I:** TWEAK 20ng/mL for 4-hours was used as a positive control for nuclear translocation; **J-L:** gentamicin (GM) 150μM, **M-O:** GM+Qx28; **P-R:** neomycin (Neo) 250μM; **S-U:** Neo+Qx28; **V-X:** CDDP 50μM; **Y-Aa:** CDDP +Qx28. Cells were fixed and immunostained for p52 (green) and counterstained with phalloidin (red). Representative images from 2 independent experiments. Scale bar = 55μm.

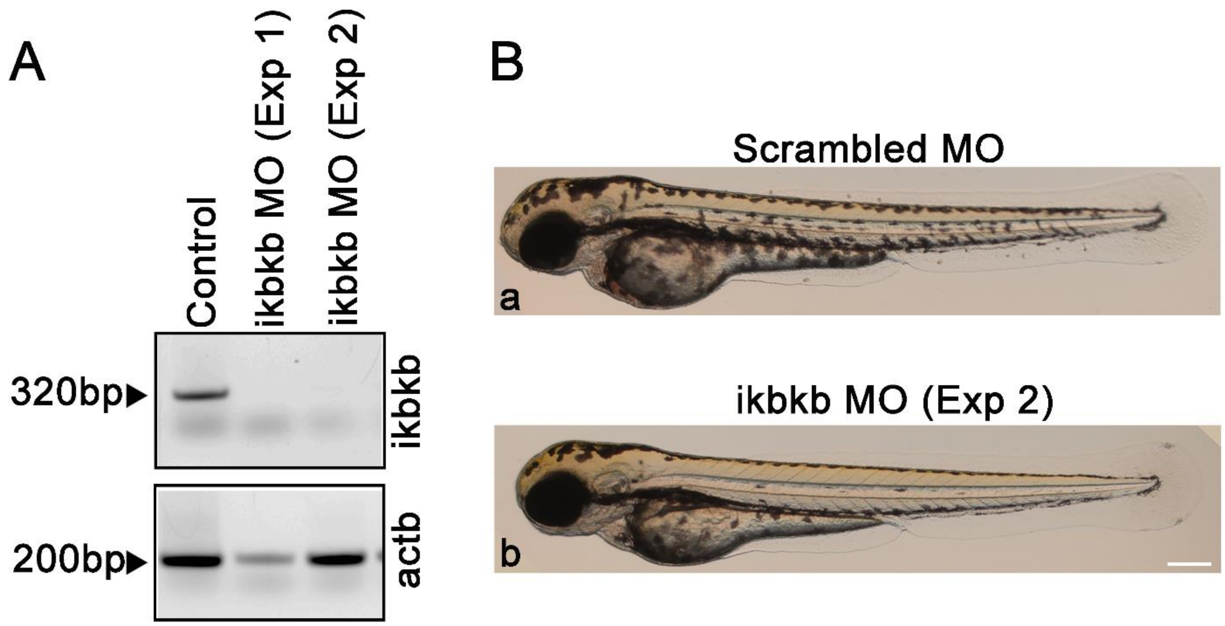

Figure S4. *ikbkb morphants showed specific ikbkb knockdown and normal gross morphology.* **A:** Four micrograms of RNA from controls (non-injected or scrambled) and *ikbkb* morphants (two independent experiments), were used in RT-PCR experiments using specific primers for *ikbkb* and *actb* (loading control). **B:** 3dpf scrambled (**a**) and *ikbkb* morphants (**b**). Scale bar: 200µm.

Compound ID	Substitution	Doses	Compound ID	Substitution	Doses
**Qx2**	2-NSO_2PhNH_2Na*	300µM, 1µM	**Qx37**	6-CH_2CO_2Me	300µM, 1µM
**Qx3**	2,3,6-trimethyl	300µM, 1µM	**Qx38**	6-amine	300µ,1µM, 10nM
**Qx4**	2-chloro	300µM	**Qx39**	2-carboxylic acid, 3-methyl	300µM
**Qx5**	2-bromo	300µM, 1µM	**Qx40**	2-hydroxy*	300µM, 1µM
**Qx6**	2-hydroxy, 7-chloro, 8-methoxy*	300µM	**Qx41**	2,3-dichloro	300µM
**Qx7**	2-methoxy, 7-bromo	300µM	**Qx42**	5-methyl	300µM, 1µM
**Qx8**	2-chloro, 7-trifluoromethyl	300µM	**Qx43**	2-CHO	300µM, 1µM
**Qx9**	2,3-dichloro, 6-methyl	300µM	**Qx44**	2-amine, 7-bromo	300µM, 1µM
**Qx10**	2-chloro, 6-nitro	300µM, 1µM, 10nM	**Qx45**	6-$COCH_3$	300µM, 1µM
**Qx11**	2,3-dithiol, 6-methyl*	300µM, 1µM, 10nM	**Qx46**	5-hydroxy*	300µM, 1µM
**Qx12**	2-carboxylic acid	300µM, 1µM, 10nM	**Qx47**	6- CO_2Et	300µM
**Qx13**	2,3-dimethyl, 5-amine, 6-methoxy	300µM	**Qx48**	2,3-dimethyl, 6,7-dimethoxy	300µM, 1µM
**Qx14**	2-chloro, 3-methoxy	300µM	**Qx49**	2,3-dichloro, 6-trifluoromethoxy*	300µM
**Qx15**	2,3-dichloro, 6-bromo	300µM	**Qx50**	2,3-diamine	300µM, 1µM
**Qx16**	2-chloro, 7-bromo	300µM	**Qx51**	5-fluoro, 7-bromo	300µM, 1µM
**Qx17**	2-chloro, 8-methoxy	300µM	**Qx52**	2-$NHSO_2PhMe$, 3-chloro*	300µM

**Qx18**	2,8-dichloro	300µM, 1µM	**Qx53**	5-nitro	300µM
**Qx19**	2-CO_2Et, 3-chloro*	300µM	**Qx54**	2-CO_2Me*	300µM
**Qx20**	2-chloro, 7-methyl	300µM, 1µM	**Qx55**	2-chloro, 6-fluoro	300µM
**QX21**	2,3-dichloro, 6-mehtoxy	300µM, 1µM	**Qx56**	2,3-dichloro, 6,7-difluoro	300µM
**Qx22**	2,6-dichloro	300µM, 1µM	**Qx57**	5-chloro	300µM
**Qx23**	2,3-dimethoxy	300µM, 1µM	**Qx58**	2-chloro, 6,7-dimethoxy	300µM
**Qx24**	6-2,4-thiazolidinedione	300µM	**Qx59**	5-bromo	300µM
**Qx26**	2-hydroxy, 6-bromo	300µM, 1µM	**Qx60**	2-chloro, 6-trifluoromethyl	300µM
**Qx27**	2-amine	300µM, 1µM	**Qx61**	6-CH_2OH	300µM
**Qx28**	5-carboxylic acid	300µM, 1µM, 10nM	**Qx62**	6-iodo	300µM
**Qx29**	2,3-dichloro, 6-CO_2Me	300µM	**Qx63**	6-BH_2O_2. HCl*	300µM
**Qx30**	2-chloro, 3-methyl	300µM, 1µM	**Qx64**	5-bromo, 8-chloro	300µM
**Qx31**	6-hydroxy*	300µM, 1µM	**Qx65**	2-chloro, 6-methoxy	300µM
**Qx32**	5-fluoro	300µM	**Qx66**	6-carboxylic acid	300µM, 100µM, 50µM, 1µM
**Qx33**	5-CO_2Me	300µM,	**Qx67**	5-bromo, 6-amine	300µM
**Qx34**	3-methyl, 5-carboxylic acid	300µM, 1µM	**Qx68**	6-methyl	300µM
**Qx35**	6-chloromethyl	300µM,	**Qx69**	2,5-dichloro	300µM
**Qx36**	2-carboxamide*	300µM, 1µM	**Qx70**	6-bromo	300µM`

Table S1. Quinoxaline derivatives: Library of the quinoxaline derivatives tested in this paper including the functional group substitutions at the different positions of the quinoxaline ring. * derivatives purchased from commercial sources.
